# [Tl_7_]^7–^ Clusters in Mixed Alkali Metal Thallides Cs_7.29_K_5.71_Tl_13_ and Cs_3.45_K_3.55_Tl_7_

**DOI:** 10.1021/acs.inorgchem.3c04034

**Published:** 2024-02-28

**Authors:** Vanessa F. Schwinghammer, Stefanie Gärtner

**Affiliations:** † Institute of Inorganic Chemistry, University of Regensburg, Universitätsstraße 31, 93053 Regensburg, Germany; ‡ Central Analytics, University of Regensburg, Universitätsstraße 31, 93053 Regensburg, Germany

## Abstract

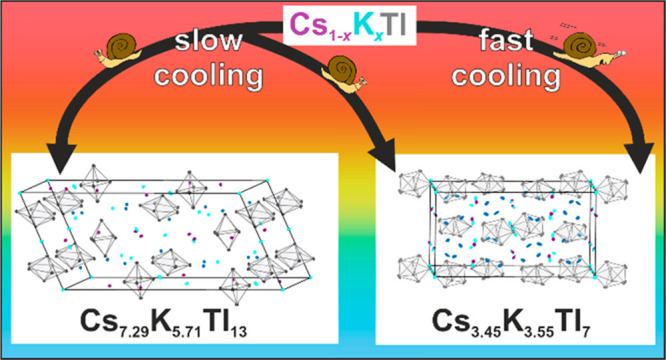

Investigations in the ternary system Cs–K–Tl resulted in the unexpected formation of new ternary thallides Cs_7.29_K_5.71_Tl_13_ and Cs_3.45_K_3.55_Tl_7_. Single crystal X-ray structure analyses of both compounds reveal the presence of isolated Tl cluster units. Cs_7.29_K_5.71_Tl_13_ crystallizes in the monoclinic space group *C*2/*c* (*a* = 30.7792(9) Å, *b* = 11.000(2) Å, *c* = 14.0291(4) Å, β = 112.676(4)°, *Z* = 4) and contains [Tl_6_]^6–^ and [Tl_7_]^7–^ clusters as thallium subunits. Cs_3.45_K_3.55_Tl_7_ crystallizes in the tetragonal space group *I*4_1_/*a* (*a* = 13.6177(2) Å, *c* = 25.5573(8) Å, *Z* = 8) and contains [Tl_7_]^7–^ clusters exclusively. The formation of Cs_7.29_K_5.71_Tl_13_ is obtained after slow cooling in addition to that of Cs_3.45_K_3.55_Tl_7_ and can be suppressed by quenching the stoichiometric mixture. First dissolution experiments in liquid ammonia suggest thallium and amide as final oxidation products. Full relativistic band structure calculations of Cs_4_K_3_Tl_7_ and Cs_8_K_5_Tl_13_ showed a (pseudo) band gap around *E*_F_ for both compounds.

## Abstract 2

Thallium clusters in mixed alkali metal ternaries! Cs_3.45_K_3.55_Tl_7_ includes [Tl_7_]^7−^, while Cs_7.29_K_5.71_Tl_13_ contains [Tl_7_]^7−^ and [Tl_6_]^6−^ clusters. Both compounds are obtained by slow cooling of the stoichiometric mixture. Quenching to room temperature suppresses the formation of Cs_7.29_K_5.71_Tl_13_.

Alkali metal thallides can be described as materials at the frontier between ionic and intermetallic nature.^1,2^ While a low amount of alkali metal of less than 50% results in the formation of rather metallic compounds,^3−7^ increasing proportions lead to the formation of salt-like materials. The observed thallium subunits can then be well described by using the Zintl–Klemm formalism.^8−11^ The first structurally characterized Zintl phase goes back to E. Zintl himself, who reported on the synthesis and characterization of textbook-known NaTl in the early 1930s.^12^ Interestingly, binaries in a 1:1 ratio (alkali metal:thallium) of the larger alkali metals were reported only in the late 1990s and 2000s by the group of J. D. Corbett. The dependence of the nature of the alkali metal involved on the formed thallium substructure is well evidenced in KTl^13^ and CsTl,^14^ which include isolated [Tl_6_]^6–^ clusters instead of the three-dimensional Tl sublattice present in NaTl.^12,15−17^ A closer look at the crystal structures of KTl and CsTl shows that the solid-state structures differ significantly, as they crystallize in different space groups (KTl, *Cmce*; CsTl, *Fddd*) and structure types. The major difference in the crystal structures of these binaries can be ascribed to one symmetry-independent alkali metal position.^18^ This suggests that potassium and cesium cannot be interchanged and emphasizes the importance of the different alkali metals in structure formation. This in general was proven for thallides in combinations of sodium and larger alkali metals in the past by Corbett et al., which showed a broad variety of new and surprising materials by applying mixed alkali metal approaches.^18^ In contrast, very little is known about the influence of mixing heavy alkali metals K and Cs. In the case of a single type of alkali metal, there are so far only five phases known that include discrete clusters. In Na_2_Tl^19^ [Tl_4_]^4–^ tetrahedra are present; *A*_8_Tl_11_ (*A* = K, Rb, Cs)^20,21^ includes the pentacapped trigonal prism [Tl_11_]^7–^, which is also known as a double tetrahedral star.^22^ In metallic K_10_Tl_7_ the pentagonal bipyramid [Tl_7_]^7–^ is present,^23^ while in the above-mentioned KTl and CsTl compressed [Tl_6_]^6–^ octahedra are observed, which have been classified as “hypoelectronic” clusters due to the lack in electrons referred to Wade electron counting rules.^13,14^ In general, [Tl_6_]^6–^ octahedra are obtained in different binary and ternary materials, e.g., *A*_10_Tl_6_O_2_ (*A* = K, Rb),^24^ Cs_10_Tl_6_*Tt*O_4_ (*Tt* = Si, Ge), or Cs_10_Tl_6_SnO_3_.^25^ Another very rare cluster unit is represented by the pentagonal bipyramidal [Tl_7_]^7–^. While heteroatomic pentagonal bipyramidal entities are known to be accessible using solution chemistry,^26,27^ the homoatomic Tl_7_ cluster represents a very rare structural moiety in the solid state. This subunit can be geometrically derived from an endohedral icosahedron by removing five vertices and is so far experimentally known only from the three compounds Na_9_K_16_Tl_∼25_,^28^ Na_12_K_38_Tl_48_Au_2_,^29^ and K_10_Tl_7_.^23^ Na_9_K_16_Tl_∼25_ and Na_12_K_38_Tl_48_Au_2_ both show crystallographic peculiarities. In Na_9_K_16_Tl_∼25_ the Tl_7_ cluster is also part of the Tl_9_ cluster, in which not all Tl positions are fully occupied.^28^ Na_12_K_38_Tl_48_Au_2_ contains Tl_7_ and Tl_9_ clusters next to each other, and isolated Au^–^ atoms are present as further anionic species.^29^ The alkali metal thallide including a Tl_7_ cluster as the exclusive thallium substructure is K_10_Tl_7_, in which three extra electrons lead to the metallic nature of the material.^23^ We report here on the synthesis, single crystal structure analysis, SEM/EDS measurements, dissolution experiment in liquid ammonia, and first band structure calculations of Cs_7.29_K_5.71_Tl_13_ and Cs_3.45_K_3.55_Tl_7_, which both include the rare [Tl_7_]^7–^ cluster.

All materials were synthesized by classical high-temperature solid-state synthesis, starting from the elements. The title compound Cs_3.45_K_3.55_Tl_7_ was first observed together with KTl and K_3.826_Cs_4.174_Tl_11_ during our systematic investigation of the mixed alkali metal system Cs_1–*x*_K_*x*_Tl in the sample with the nominal composition CsK_2_Tl_3_ (see Supporting Information (SI) section 8). The approach according to the stoichiometric composition Cs_4_K_3_Tl_7_ included both title compounds Cs_3.45_K_3.55_Tl_7_ and Cs_7.29_K_5.71_Tl_13_ next to each other and was characterized by single crystal X-ray structure analysis (Table 1, see SI sections 3 and 4 for the atomic coordinates and displacement parameters).

**Table 1:** Extract from the Crystallographic Data of Cs_7.29_K_5.71_Tl_13_ and Cs_3.45_K_3.55_Tl_7_

Empirical formula	Cs_7.29_K_5.71_Tl_13_	Cs_3.45_K_3.55_Tl_7_
CSD number	2295683	2296130
Formula weight/g mol^–1^	3849.82	2026.95
Temperature/K	123	
Crystal system, space group	monoclinic, *C*2/*c*	tetragonal, *I*4_1_/*a*
*a*/Å	30.7792(9)	13.6177(2)
*b*/Å	11.0000(2)	=*a*
*c*/Å	14.0291(4)	25.5573(8)
β/deg	112.676(4)	90
Volume/Å^3^, *Z*	4382.7(2), 4	4739.39(18), 8
Radiation	Ag Kα (λ = 0.56087 Å)	
ρ_calc_/(g cm^–^^3^)	5.835	5.681
μ/mm^–1^	29.146	28.681
*R*_int_	0.0577	0.0387
Final *R* indexes [*I* ≥ 2σ(*I*)]	*R*_1_/*wR*_2_ = 0.0390/0.0650	*R*_1_/*wR*_2_ = 0.0356/0.0530
Final *R* indexes [all data]	*R*_1_/*wR*_2_ = 0.0514/0.0685	*R*_1_/*wR*_2_ = 0.0554/0.0587

The powder diffraction analysis of the bulk material showed additionally K_3.79_Cs_4.21_Tl_11_ being present (see SI Figure S15). The formation of the favored *A*_8_Tl_11_ is commonly known to prevent phase purity of several thallide materials.^20,21^ Binary CsTl or KTl could not be indexed. Further Cs_1–*x*_K_*x*_Tl approaches, also with a modified temperature program (quenching to room temperature), always resulted in the formation of a mixture of Cs_3.45_K_3.55_Tl_7_ and/or Cs_7.29_K_5.71_Tl_13_ (see Table 2 and SI section 8). Generally, Cs_8–*x*_K_*x*_Tl_11_ is present as a side phase. Concerning the title compounds a trend is discernible. While Cs_3.45_K_3.55_Tl_7_ is formed ubiquitously and independent from the temperature program applied, slow cooling additionally enables the formation of Cs_7.29_K_5.71_Tl_13_. Vice versa, this can be suppressed, as upon quenching only a mixture of Cs_8–*x*_K_*x*_Tl_11_ and Cs_3.45_K_3.55_Tl_7_ is formed. The exact ratio of potassium and cesium and even a small excess of alkali metal does not influence this observation, as the temperature program is crucial. SEM/EDS measurements of the metallic dark gray blocks support the composition of the single crystals (see SI section 12).

**Table 2:** Formation of Cs_3.45_K_3.55_Tl_7_ and Cs_7.29_K_5.71_Tl_13_ in Cs_1–*x*_K_*x*_Tl Samples Depends on the Temperature Program Applied^a^

	Cooling rate			
Samples	5 K/h	Quenching	Cs_3.45_K_3.55_Tl_7_	Cs_7.29_K_5.71_Tl_13_
Cs_2_KTl_3_	■		■	■
CsK_2_Tl_3_	■		■	■
CsKTl_2_	■		■	■
CsKTl_2_		■	■	
Cs_4_K_3_Tl_7_	■		■	■
Cs_3.45_K_3.55_Tl_7_	■		■	■
Cs_3.45_K_3.55_Tl_7_		■	■	
Cs_7.29_K_5.71_Tl_13_		■	■	
Cs_7.56_K_6.0_Tl_13_	■		■	■
Cs_7.56_K_6.0_Tl_13_		■	■	

a Cs_8–*x*_K_*x*_Tl_11_ is always present. Related PXRD are given in SI section 8.

Since a 1:1 ratio of alkali metals:thallium was applied, the occurrence of [Tl_6_]^6–^ octahedra was expected as these clusters are observed in KTl and CsTl. This is indeed the case for Cs_7.29_K_5.71_Tl_13_, but in addition to [Tl_6_]^6–^, [Tl_7_]^7–^ pentagonal bipyramids are also present in the crystal structure of the latter. In the unit cell of Cs_3.45_K_3.55_Tl_7_, [Tl_7_]^7–^ clusters as the anionic moiety are exclusively present.

The compound Cs_7.29_K_5.71_Tl_13_ crystallizes in the monoclinic space group *C*2/*c*. The asymmetric unit consists of seven thallium and seven alkali metal atoms. The alkali metal sites are not randomly mixed occupied but can be subdivided into two cesium, three potassium, and two atomic sites mixed occupied by cesium and potassium. The [Tl_6_]^6–^ octahedron is built by the thallium atoms Tl1–Tl3 (Wyckoff position 8*f*), and the pentagonal bipyramid [Tl_7_]^7–^ is built by Tl4–Tl7 (Wyckoff position 8*f*/4*e* (Tl7)) (see Figure 1).

**Figure 1:**
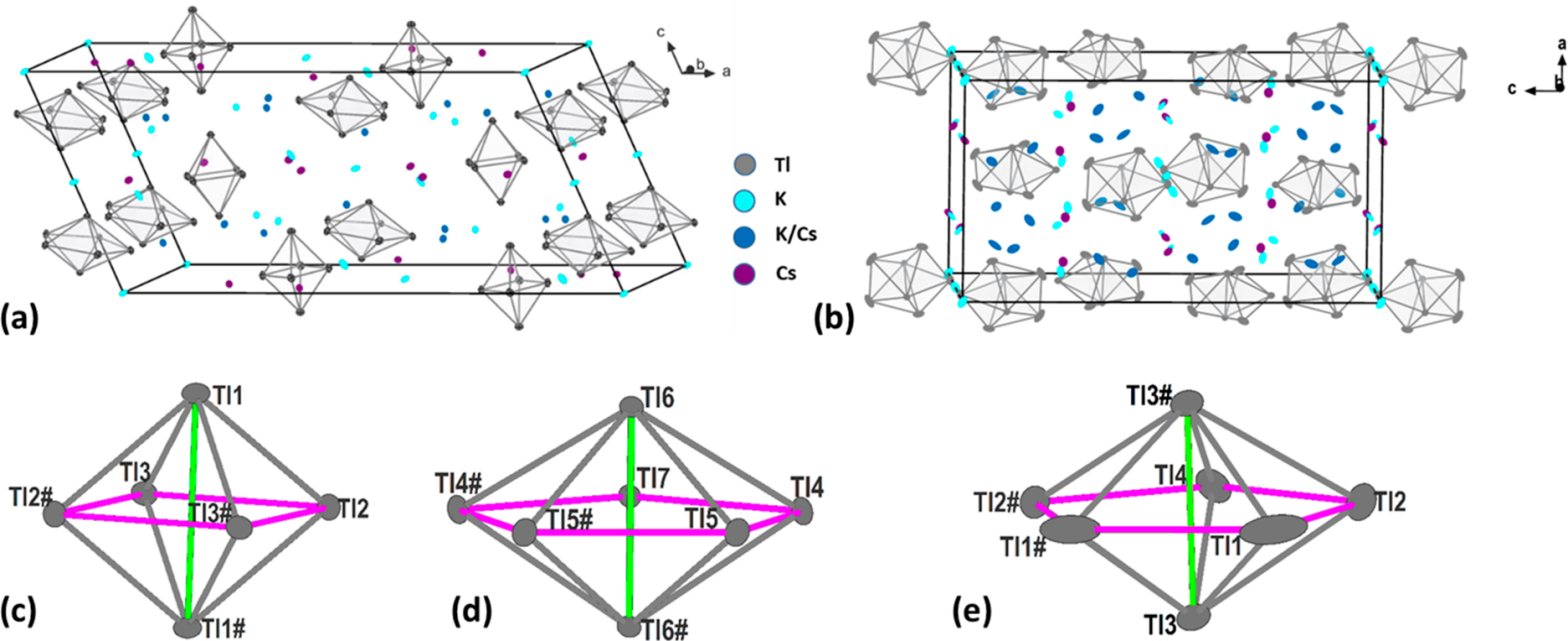
Unit cells of Cs_7.29_K_5.71_Tl_13_ (a) and Cs_3.45_K_.3.55_Tl_7_ (b) with the potassium atoms depicted in light blue, the mixed occupied atoms in dark blue, and the cesium atoms in purple. The [Tl_6_]^6—^ octahedron (c) (Tl atoms labeled with # are at equivalent positions (1/2 – *x*, 1/2 – *y*, 1 – *z*)) and pentagonal bipyramids [Tl_7_]^7—^ (d) (Tl atoms labeled with # are at equivalent positions (1 – *x*, *y*, 1/2 – *z*)) in Cs_7.29_K_5.71_Tl_13_ and (e) (Tl atoms labeled with # are at equivalent positions (1 – *x*, 1/2 – *y*, *z*)) in Cs_3.45_K_3.55_Tl_7_. The equatorial Tl–Tl distance is marked in pink, and the distances between the apical atoms (Tl1–Tl1 or Tl6–Tl6) are marked in green.

The second compound Cs_3.45_K_3.55_Tl_7_ crystallizes tetragonal in the space group *I*4_1_/*a* and is formed by four crystallographically independent thallium atoms (Wyckoff sites 16*f*/8*e* (Tl7)), which form the pentagonal bipyramid (Figure 1b). Four alkali metal atoms complete the asymmetric unit and can be subdivided into one potassium (Wyckoff site 8*c*), two mixed occupied atomic sites, and one split position of cesium and potassium (Wyckoff sites 16*f*). For Cs_3.45_K_3.55_Tl_7_ the arrangements of the clusters can be described as hexagonal layers arranged in an AB stacking sequence of a distorted αU packing (see SI Figure S16). For comparison, according to Corbett et al. the clusters in K_10_Tl_7_ can be described as hcp packed.^23^ A detailed structural description of both compounds can be found in section 6 of the SI.

In order to compare Tl_6_ and Tl_7_ clusters, respectively, a ratio  of the averaged distances of the equatorial atoms (*d*_eq_, pink distances in Figure 1c–e) and the distance of the apical atoms (*d*_ap_, green distances in Figure 1c–e) can be calculated.^23^ In an uncompressed octahedron, the value would calculate to *sqrt*(2) ≈ 1.414. Due to the axial compression, the [Tl_6_]^6–^ octahedra in general show smaller values between 0.98 and 1.16 (see SI Table S10). The values of [Tl_7_]^7–^ calculate also to a slightly smaller range of 1.02–1.08 (see SI Table S11). An uncompressed *closo*-Tl_7_ cluster would afford a 9-fold negative charge in analogy to *closo*-2,4-C_2_B_5_H_7_. Axial compression of these Tl_7_ clusters in K_10_Tl_7_ was reported as a result of the “*hypoelectronic*” nature of [Tl_7_]^7–^.^23^ For K_10_Tl_7_, Corbett et al. suggested a 7-fold negative charge by analyzing extended Hückel molecular orbital (EHMO) calculations. Detailed theoretical investigations of the corresponding solid-state structure by Jansen et al. showed a pseudo band gap below *E*_F_.^30^ The integrated DOS between this gap and the Fermi level amounts to three electrons, which supports a [Tl_7_]^7–^ cluster. As the compounds reported here should be electronically balanced, we calculated the total DOS (for details see SI S6, 2.4). Mixed occupied sites are difficult to address in band structure calculations as an enlargement of the unit cell results in intolerable expensive calculations for these heavy atom structures. Therefore, we assumed fully occupied sites for our first band structure calculations to get insight into the electronic nature of the new compounds. Both materials show a (pseudo) band gap around *E*_F_ (for Cs_4_K_3_Tl_7_ it is left and for Cs_8_K_5_Tl_13_ right of *E*_F_; slight deviations must be addressed in detailed calculations), which suggests the classification of a Zintl phase assuming complete electron transfer from the less electronegative alkali metals to thallium (Figure 2). The compounds reported here therefore yield a charge of −7 for the Tl_7_ cluster and emphasize the packing effects on the obtained cluster compounds, as binary K_7_Tl_7_ cannot be obtained.

**Figure 2:**
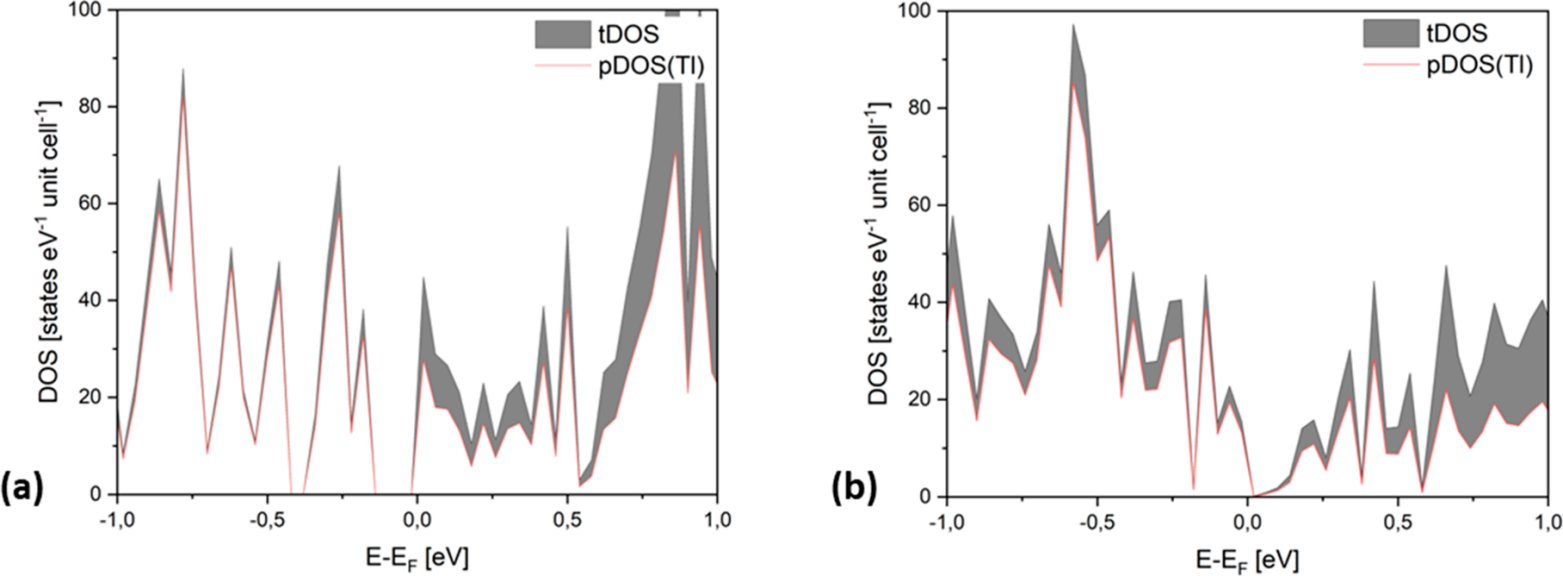
Total DOS (gray) and partial DOS of Tl (white) of the theoretical compounds Cs_4_K_3_Tl_7_ (a) and Cs_8_K_5_Tl_13_ (b).

Salt-like Zintl phases including isolated clusters are of great interest in material science, as they provide p-block metal building blocks,^31^ which can be transferred and altered using solution chemistry methods.^32−40^ For homoatomic clusters, this is proven for groups 14–16. In contrast, no group 13 cluster has yet been obtained from dissolving trielide Zintl phases. Because of the relatively low charge of the group 13 clusters [Tl_6_]^6–^ and [Tl_7_]^7–^, these materials might be appropriate representatives. Therefore, first dissolution experiments in liquid ammonia were carried out, which yielded thallium and alkali metal amide as final oxidation products (SI section 11). Similar observations have been made for Na_8–*x*_*A*_*x*_Tl_4_ (*A* = K, Rb; *x* = 0, 1).^41,42^

The occurrence of different clusters for isoelectronic thallide atoms in [Tl_*x*_]^*x*−^ (*x* = 6, 7), even within one crystal structure, emphasizes the filigree interplay of packing effects and stabilization of the thallide clusters. The simultaneous application of different heavier congeners of the alkali metals within one sample seems to provide new and unpredictable compounds for this class of materials. These mixed cation approaches allow the formation of electronically balanced clusters precast in salt-like Zintl phases. The homoatomic group 13 clusters might be appropriate representatives for investigating solution chemistry in the style of group 14 or 15 Zintl phases. Ongoing experiments will show whether it is possible to transfer homoatomic trielide clusters from solid state into solution or if this is limited to clusters right to the Zintl border.

## Supporting Information Available

Experimental and DFT calculation details, crystallographic data, additional structure description, powder diffraction patterns, and dissolution experiment in liquid ammonia (PDF)

## Notes

***Caution! The element thallium as well as compounds including it are highly toxic. The alkali metals are strongly reactive. The resulting products are very sensitive to air and moisture***.
