# Supplementary material for: [Tl7]7– Clusters in Mixed Alkali Metal Thallides Cs7.29K5.71Tl13 and Cs3.45K3.55Tl7
Source: Inorg Chem. 2024 Feb 28;63(43):20078–82. doi: 10.1021/acs.inorgchem.3c04034 (PMC11523231; doi:10.1021/acs.inorgchem.3c04034)
Supplement: Supplementary file 1 — ic3c04034_si_001.pdf [file ic3c04034_si_001.pdf]

Supporting Information

$[Tl_7]^{7-}$ Clusters in Mixed Alkali Metal Thallides

$Cs_{7.29}K_{5.71}Tl_{13}$ and $Cs_{3.45}K_{3.55}Tl_7$

Vanessa F. Schwinghammer(a), Stefanie Gärtner*(a,b)

(a) Institute of Inorganic Chemistry, University of Regensburg, Universitätsstraße 31, 93053 Regensburg
(b) Central Analytics, University of Regensburg, Universitätsstraße 31, 93053 Regensburg

* Email: Stefanie.Gaertner@ur.de, Phone: +49 943 941 4446

Content

1. Crystallographic Data 4

2. Experimental 5

2.1 Materials and Synthesis 5

2.2 X-Ray Single Crystal Analysis 5

2.3 Powder Diffraction Studies 6

2.4 DFT Calculations 6

2.5 SEM/EDS 6

2.6 Dissolution Experiments in Liquid Ammonia 6

3. Atomic Coordinates 7

3.1 Atomic Coordinates of $Cs_{7.29}K_{5.71}Tl_{13}$ 7

3.2 Atomic Coordinates of $Cs_{3.45}K_{3.55}Tl_7$ 8

4. Displacement Parameter 9

4.1 Displacement Parameter of $Cs_{7.29}K_{5.71}Tl_{13}$ 9

4.2 Displacement Parameters of $Cs_{3.45}K_{3.55}Tl_7$ 9

5. Distances 10

5.1 Selected Atomic Distances in $Cs_{7.29}K_{5.71}Tl_{13}$ 10

5.2 Selected Atomic Distances in $Cs_{3.45}K_{3.55}Tl_7$ 11

6. Supplementary Structure Description 12

6.1 $Cs_{7.29}K_{5.71}Tl_{13}$ 12

6.2 $Cs_{3.45}K_{3.55}Tl_7$ 13

7. Cluster Distances in Comparison to Literature-Known Compounds 14

7.1 Distances of Compressed $[Tl_6]^{6-}$ Octahedra 14

7.2 Distances of Pentagonal Bipyramids $[Tl_7]^{7-}$ 14

8. Experimental Solid State Approaches 15

8.1 Powder Diffraction Pattern of the Approach Cs_2KTl_3 16

.... 16

8.2 Powder Diffraction Pattern of the Approach CsK_2Tl_3 16

8.3 Powder Diffraction Pattern of the Approach $CsKTl_2$ (Temp. 1) 17

8.4 Powder Diffraction Pattern of the Approach $CsKTl_2$ (Temp. 2) 17

.... 18

8.5 Powder Diffraction Pattern of the Approach $Cs_{3.45}K_{3.55}Tl_7$ (Temp. 1) 18

8.6 Powder Diffraction Pattern of the Approach $Cs_{3.45}K_{3.55}Tl_7$ (Temp. 2) 18

8.7 Powder Diffraction Pattern of the Approach $Cs_{7.29}K_{5.71}Tl_{13}$ 19

8.8 Powder Diffraction Pattern of the Approach $Cs_{7.65}K_6Tl_{13}$ (Temp. 1) 20

8.9 Powder Diffraction Pattern of the Approach $Cs_{7.65}K_6Tl_{13}$ (Temp. 2) 20

8.10 Powder Diffraction Pattern of the Approach $Cs_4K_3Tl_7$ 21

9. Packing Order of the $[Tl_7]^{7-}$ Cluster in $Cs_{3.45}K_{3.55}Tl_7$ 22
10. Band Structure 23
11. Dissolution Experiments in Liquid Ammonia 24
12. SEM/EDS Measurements 25
12.1 Measurements of the Approach K_2CsTl_3 25
12.2 Measurements on the Approach $Cs_4K_3Tl_7$ 27
13. Literature 28

1. Crystallographic Data

Table S1: Crystallographic data of the title compounds $Cs_{7.29}K_{5.71}Tl_{13}$ and $Cs_{3.45}K_{3.55}Tl_7$.

Empirical formula	**$Cs_{7.29}K_{5.71}Tl_{13}$**	**$Cs_{3.45}K_{3.55}Tl_7$**
CSD number	2295683	2296130
Formular weight	3849.818	2026.951
Temperature /K	123	
Crystal system	monoclinic	tetragonal
Space group	*C*2/*c*	*I*4$_1$/*a*
a /Å	30.7792(9)	13.6177(2)
b /Å	11.0000(2)	=*a*
c /Å	14.0291(4)	25.5573(8)
β /°	112.676(4)	90
Volume /Å^3	4382.7(2)	4739.39(18)
Z	4	8
Radiation	Ag Kα (λ=0.56087 Å)	
ρ_{calc} /gcm^{-3}	5.835	5.681
μ /mm^{-1}	29.146	28.681
F(000)	6201.4	6540.4
Crystal size /mm^3	0.058 x 0.056 x 0.056	0.08 x 0.06 x 0.04
2θ range for data collection /°	4.26 to 61.32	4.46 to 61.3
Index ranges	-55≤h≤55<br>-19≤k≤20<br>-25≤l≤25	-24≤h≤18<br>-23≤k≤24<br>-46≤l≤46
Reflections independent/collected	13791/66486	7457/47088
Data/restraints/parameter	13791/0/122	7457/0/141
Goodness-of-fit on F^2	1.052	1.046
R_{int}	0.0577	0.0387
Final R indexes $[I \geq 2\sigma(I)]$	R_1/wR_2=0.0390/0.0650	R_1/wR_2=0.0356/0.0530
Final R indexes [all data]	R_1/wR_2=0.0514/0.0685	R_1/wR_2=0.0554/0.0587
Largest diff. peak/hole /eÅ^{-3}	4.85/-3.58	5.66/-4.61

2. Experimental

2.1 Materials and Synthesis

Potassium (purity 99%, Merck/Sigma-Aldrich, Darmstadt) was segregated for purification. Cesium was obtained by reduction of CsCl with elemental calcium and afterward purified by two times distillation.[1] Thallium drops (ABCR, 99.999%) were used without further purification and were stored under an inert gas atmosphere.

The different approaches (see Chapter 8) were generated by placing the elements in tantalum ampoules, which were afterwards sealed (argon atmosphere). The sealed ampoules were placed in quartz glass tubes (QSIL GmbH, Ilmenau, Germany), which were also sealed (argon atmosphere). The following temperature programs were used:

1: heating up to 773.15 K with a heating rate of 100 K/h, which was held for 48 h, cooling to room temperature with a cooling rate of 5 K/h.

2: heating up to 773.15 K with a heating rate of 100 K/h, which was held for 48 h and then quenching to room temperature with water.

Due to the sensitivity to moisture and air, the received product was stored in a glovebox (Labmaster 130 G, Fa. M. Braun, Garching, Germany).

2.2 X-Ray Single Crystal Analysis

Suitable crystals were isolated from small amounts of the product in dried mineral oil. The crystals were mounted on a Rigaku SuperNova diffractometer (Rigaku Polska sp. Z. o. o. UI, Wroclaw, Poland) (X-ray: Ag microfocus, Atlas S2 detector) using MiTeGen loops. All data was collected at 123 K.

For data collection and data reduction, the software *CrysAlisPro* (version 171.42.84a) was used.[2] In the program *Olex²* the structure solution with *ShelXT* was carried out and for the data refinement of $Cs_{7.29}K_{5.71}Tl_{13}$ *ShelXL* and for $Cs_{3.45}K_{3.55}Tl_7$ *olex2refine* was used.[3-7] The latter compound showed a prolate ellipsoid for Tl1. Initially, it seemed to be disordered into three split positions with distances smaller than 0.35 Å. However, measurements of the same crystal at 100 K and 200 K showed no change in the size or displacement of the atom. This led to the assumption of a rotating movement of the pentagon of the pentagonal bipyramid. Subsequently, anharmonic refinement of the equatorial thallium atoms was applied.[8] For all thallium atoms beside Tl3 Kuhs rule is fullfilled (for Tl 3 a higher resolution would be required).[9] This and the significant values of the third and fourth order refinement suggest that anharmonic refinement is appropriate.

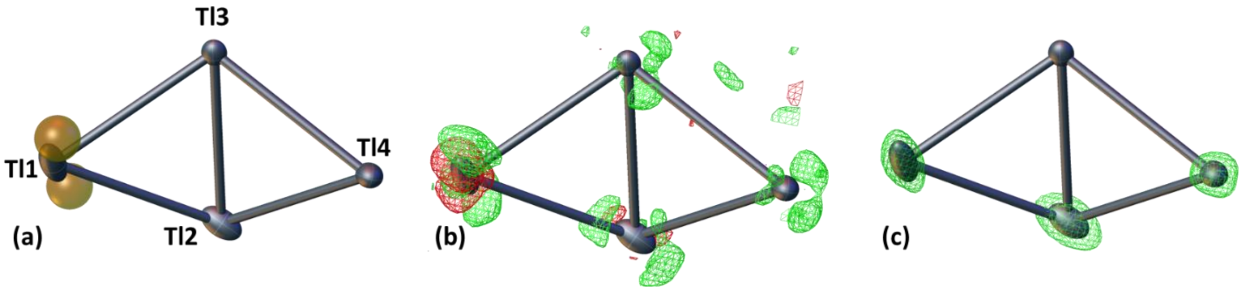

Figure S1: Refinement progress of the pentagonal bipyramid $[Tl_7]^{7-}$ in $Cs_{3.45}K_{3.55}Tl_7$ in the program Olex², where first a split position was considered (a), but then a rotation in the plane of the equatorial thallium positions was realized (b) and applied (c).

Large residual electron density around heavy atoms and large weighting factors are common for the refinement of such compounds. The very good data of a very well-grown single crystal of the

compound $Cs_{3.45}K_{3.55}Tl_7$ with a comparably small unit cell allowed for improving these issues by applying anharmonic refinement.

All figures from the crystal structure were visualized with the software *Diamond4*.[10] All atoms are depicted as ellipsoids with a 50% probability level.

2.3 Powder Diffraction Studies

The powder diffraction sample were prepared in a sealed capillary (Ø0.3 mm, WJM-Glas-Müller GmbH, Berlin, Germany). The data collection was carried out on a STOE Stadi P diffractometer (STOE, Darmstadt, Germany) (Monochromatic MoKα radiation, ʎ=0.70926 Å) equipped with a Dectris Mythen 1K detector. For visualisation and indexation the softwares *WinXPOW* and *Jana2006* were used.[11, 12]

2.4 DFT Calculations

Theoretical calculations were performed on the hypothetical compounds $Cs_8K_5Tl_{13}$ and $Cs_4K_3Tl_7$ with fully occupied alkali metal positions. The program *FPLO21*[13-16] was used, which is based on the full-potential non orthogonal local orbital minimum-basis within the generalized gradient approximation (GGA) for a full-relativistic mode. The exchange correlation was assumed in the form proposed by Perdew, Burke and Ernzerhof (PBE).[17] For the calculation of the density of states (DOS) and the band structure a modular grid for the reciprocal space of 216 k-points was sufficient. As convergence criterion a change of the total energy ($\Delta E_{tot} \leq 10^{-6}\ Hartree$) was applied. For the visualisation of the DOS the program *Origin2022* (version 9.9.0.225) was used.[18]

2.5 SEM/EDS

The crystals for the SEM/EDS measurements were prepared and selected in a glove box under inert gas atmosphere. The Measurements were performed on a Zeiss EVO MA15 (Carl Zeiss Microscopy Deutschland GmbH, Oberkochen) using the software SmartSEM Version 6.05 with accelerating voltage of 20 kV. For EDS measurements a Bruker Quantax 200-Z3 Xflash630 (Bruker, Corporation, Billerica, USA) was used as X-ray detector with the software Bruker Esprit 2.1.2.

2.6 Dissolution Experiments in Liquid Ammonia

The experiments were carried out in *Schlenk* flasks, which were flame-dried three times before adding a small amount of the product. After that, dry liquid ammonia was condensed at about 195 K on the products using *Schlenk* technique. After storing the vessel at 233 K for five months, liquid ammonia was evaporated. The residue was removed from the Schlenk in the glove box and a small amount was used in a glass capillary (0.3mm) for powder diffraction investigations.

3. Atomic Coordinates

3.1 Atomic Coordinates of $Cs_{7.29}K_{5.71}Tl_{13}$

Table S2: Fractional Atomic Coordinates and Equivalent Isotropic Displacement Parameters (Å^2×10^3) for $Cs_{7.29}K_{5.71}Tl_{13}$. U_{eq} is defined as 1/3 of the trace of the orthogonalised U_{IJ} tensor. The structure was standardized with the program Structure Tidy[19-21] implemented in Platon[22-24].

Atom	*x*	*y*	*z*	U(eq)
Tl1	0.187308(7)	0.295087(18)	0.489224(16)	0.01739(4)
Tl2	0.222593(7)	0.247613(17)	0.317312(15)	0.01692(4)
Tl3	0.233018(7)	0.039435(17)	0.500969(16)	0.01631(4)
Tl4	0.422526(7)	0.337244(17)	0.286369(16)	0.01593(4)
Tl5	0.451997(7)	0.068744(16)	0.271818(15)	0.01587(4)
Tl6	0.467337(6)	0.259753(16)	0.122139(14)	0.01319(3)
Tl7	0	0.00880(2)	0.2500	0.01271(4)
Cs1	0.054118(14)	0.37739(4)	0.46112(3)	0.02348(7)
Cs2	0.087340(14)	0.24245(3)	0.19535(3)	0.01884(11)
Cs3	0.172742(13)	0.30865(3)	0.00121(3)	0.02291(7)
Cs4	0.323423(15)	0.07312(4)	0.26240(3)	0.01965(11)
K1	0.087340(14)	0.24245(3)	0.19535(3)	0.01884(11)
K2	0.11377(5)	0.02699(12)	0.43739(12)	0.0275(3)
K3	0.30685(4)	0.44151(10)	0.26862(9)	0.01704(19)
K4	0.323423(15)	0.07312(4)	0.26240(3)	0.01965(11)
K5	0	0	0	0.0219(3)

3.2 Atomic Coordinates of $Cs_{3.45}K_{3.55}Tl_7$

Table S3: Fractional Atomic Coordinates and Equivalent Isotropic Displacement Parameters (Å^2×10^3) for $Cs_{3.45}K_{3.55}Tl_7$. U_{eq} is defined as 1/3 of the trace of the orthogonalised U_{IJ} tensor. The structure was standardized with the program cif2standard implemented in the Bilbao Crystallographic Server[25-27].

Atom	*x*	*y*	*z*	U(eq)
Tl1	0.60208(6)	0.69196(5)	0.30505(3)	0.0560(3)
Tl2	0.66387(3)	0.65649(4)	0.42453(2)	0.03530(15)
Tl3	0.562257(13)	0.856656(13)	0.388867(7)	0.02026(4)
Tl4	0.5000	0.7500	0.49601(2)	0.02716(15)
Cs1	0.67340(9)	0.67105(8)	0.16940(5)	0.0461(4)
Cs2	0.62061(4)	0.42511(5)	0.34773(3)	0.0491(2)
Cs3	0.78794(4)	0.87223(6)	0.49466(2)	0.03312(17)
K3	0.7803(8)	0.8267(15)	0.5000(4)	0.034(2)
K4	0.5000	0.5000	0.5000	0.0344(4)
K2	0.62061(4)	0.42511(5)	0.34773(3)	0.0491(2)
K1	0.67340(9)	0.67105(8)	0.16940(5)	0.0461(4)

4. Displacement Parameter

4.1 Displacement Parameter of $Cs_{7.29}K_{5.71}Tl_{13}$

Table S4: Anisotropic Displacement Parameters for $Cs_{7.29}K_{5.71}Tl_{13}$. The Anisotropic displacement factor exponent takes the form: $-2\pi^2[h^2a^{*2}U_{11}+2hka^*b^*U_{12}+\ldots]$.

Atom	U_{11}	U_{22}	U_{33}	U_{12}	U_{13}	U_{23}
Tl1	0.01214(8)	0.01892(8)	0.02150(9)	-0.00149(6)	0.00692(7)	-0.00193(6)
Tl2	0.01846(9)	0.01719(8)	0.01426(8)	-0.00101(6)	0.00535(7)	-0.00012(6)
Tl3	0.01450(8)	0.01387(7)	0.01951(9)	-0.00169(6)	0.00541(7)	-0.00047(6)
Tl4	0.01148(7)	0.01741(8)	0.01997(9)	0.00158(6)	0.00723(7)	0.00137(6)
Tl5	0.01778(8)	0.01370(7)	0.01829(8)	-0.00210(6)	0.00933(7)	0.00076(6)
Tl6	0.01236(7)	0.01477(7)	0.01107(7)	-0.00109(5)	0.00300(6)	0.00118(5)
Tl7	0.01148(10)	0.01347(10)	0.01274(10)	0	0.00417(8)	0
Cs1	0.02242(17)	0.03010(18)	0.01841(16)	0.00498(13)	0.00841(13)	0.00549(13)
Cs2	0.01665(18)	0.01765(17)	0.0223(2)	-0.00122(11)	0.00758(14)	-0.00078(12)
Cs3	0.02121(16)	0.02655(17)	0.02110(17)	-0.00669(12)	0.00828(14)	-0.00129(13)
Cs4	0.01747(19)	0.02100(19)	0.0211(2)	-0.00350(13)	0.00809(15)	-0.00406(14)
K1	0.01665(18)	0.01765(17)	0.0223(2)	-0.00122(11)	0.00758(14)	-0.00078(12)
K2	0.0155(5)	0.0228(6)	0.0348(7)	-0.0048(4)	-0.0008(5)	-0.0001(5)
K3	0.0156(5)	0.0177(5)	0.0191(5)	0.0014(3)	0.0081(4)	0.0034(4)
K4	0.01747(19)	0.02100(19)	0.0211(2)	-0.00350(13)	0.00809(15)	-0.00406(14)
K5	0.0307(9)	0.0227(8)	0.0149(7)	-0.0023(6)	0.0118(7)	0.0037(5)

4.2 Displacement Parameters of $Cs_{3.45}K_{3.55}Tl_7$

Table S5: Anisotropic Displacement Parameters for $Cs_{3.45}K_{3.55}Tl_7$. The Anisotropic displacement factor exponent takes the form: $-2\pi^2[h^2a^{*2}U_{11}+2hka^*b^*U_{12}+\ldots]$.

Atom	U_{11}	U_{22}	U_{33}	U_{12}	U_{13}	U_{23}
Tl1	0.0793(6)	0.0467(4)	0.0419(4)	-0.0405(4)	0.0344(4)	-0.0245(3)
Tl2	0.0204(2)	0.0300(3)	0.0556(4)	0.00211(18)	-0.0046(2)	-0.0144(2)
Tl3	0.02064(8)	0.01999(8)	0.02016(8)	-0.00625(6)	0.00169(6)	-0.00205(6)
Tl4	0.0380(4)	0.0200(3)	0.0236(3)	0.0038(3)	0	0
Cs1	0.0599(8)	0.0323(5)	0.0461(7)	-0.0178(4)	0.00238(5)	-0.0074(4)
Cs2	0.0319(3)	0.0463(3)	0.0692(5)	0.0117(2)	-0.0115(3)	-0.0394(3)
Cs3	0.0280(2)	0.0404(4)	0.0310(3)	0.0000(2)	-0.01145(17)	-0.0007(2)
K3	0.028(5)	0.053(7)	0.021(4)	-0.004(5)	-0.016(3)	0.003(5)
K4	0.0502(11)	0.0199(7)	0.0332(9)	-0.0047(7)	-0.0166(8)	0.0067(6)
K2	0.0319(3)	0.0463(3)	0.0692(5)	0.0117(2)	-0.0115(3)	-0.0394(3)
K1	0.0599(8)	0.0323(5)	0.0461(7)	-0.0178(4)	0.0238(5)	-0.0074(4)

5. Distances

5.1 Selected Atomic Distances in $Cs_{7.29}K_{5.71}Tl_{13}$

Table S6: Selected atomic distances of the first coordination sphere in $Cs_{7.29}K_{5.71}Tl_{13}$. The numbering scheme of the atoms is according to Figure 1 and Figure S3.

**Tl1 (8*f*)**		**Tl2 (8*f*)**		**Tl3 (8*f*)**	
Tl1	3.8819(4)	Tl3	3.3418(3)	Tl3	4.7513(4)
			3.3687(3)		
Tl2	3.0477(3)	Cs2/K1	3.8431(5)	Cs3	3.8637(4)
	3.0780(3)				4.2553(4)
Tl3	3.0143(3)	Cs3	4.1501(5)	Cs4/K4	3.8547(5)
	3.1203(3)				
Cs1	4.0663(4)	Cs4/K4	3.8543(4)	K2	3.4294(14)
			3.9689(5)		
Cs2/K1	4.1254(5)	K3	3.5735(11)	K3	3.6595(12)
			3.6204(11)		3.8804(12)
Cs3	4.3915(4)				
Cs4/K4	3.9038(5)				
	4.5854(5)				
K2	3.6165(13)				
K3	4.2280(12)				
**Tl4 (8*f*)**		**Tl5 (8*f*)**		**Tl6 (8*f*)**	
Tl5	3.1194(3)	Tl5	3.2406(4)	Tl6	3.3692(4)
Tl6	3.2230(3)	Tl6	3.1323(3)	Tl7	3.2161(3)
	3.2421(3)		3.1535(3)		
Tl7	3.2320(2)	Cs1	3.8303(4)	Cs1	4.3469(4)
			3.8635(4)		4.3778(8)
			3.687(4)		
Cs1	4.0837(4)	Cs2/K1	3.8718(4)	Cs2/K1	4.1100(5)
Cs2/K1	4.4816(4)	Cs3	4.4601(5)	Cs3	4.0493(4)
Cs3	4.2813(5)	Cs4/K4	3.9087(5)	K2	3.7368(13)
Cs4/K4	4.1313(4)			K5	3.50129(18)
K2	3.5714(15)				
K3	3.6565(12)				
K5	3.5162(2)				
**Tl7 (4*e*)**		**Cs1 (8*f*)**		**Cs2/K1 (8*f*)**	
Cs1	4.9175(5)	Cs1	4.7293(7)	Cs3	4.5142(9)
Cs2/K1	4.0011(4)	Cs2/K1	4.3139(6)	K2	3.9596(16)
			4.4822(6)		4.9815(16)
K2	3.4808(13)	Cs3	4.8977(7)	K3	4.5333(12)
K5	3.50861(10)	Cs4/K4	4.2749(6)	K5	4.0211(4)
		K2	4.3370(15)		
		K5	4.5832(5)		
**Cs3 (8*f*)**		**Cs4/K4 (8*f*)**		**K2 (8*f*)**	
Cs3	4.9405(8)	K3	4.0893(12)	K3	3.9154(19)
			4.1253(12)		4.5443(22)
Cs4/K4	3.9648(7)			K5	3.9363(15)
	4.3785(6)				
K2	4.0589(14)				
K3	4.6158(11)				
	4.9127(14)				
	5.0564(12)				

5.2 Selected Atomic Distances in $Cs_{3.45}K_{3.55}Tl_7$

Table S7: Selected atomic distances of the first coordination sphere in $Cs_{3.45}K_{3.55}Tl_7$. The numbering scheme of the atoms is according to Figure 1 and Figure S5.

**Tl1 (16*f*)**		**Tl2 (16*f*)**		**Tl3 (16*f*)**	
Tl1	3.1981(17)	Tl3	3.1899(5)	Tl3	3.3635(3)
			3.2164(5)		
Tl2	3.2041(10)	Tl4	3.1526(6)	Tl4	3.2136(6)
Tl3	3.1485(6)	Cs1/K1	3.6697(11)	Cs1/K1	3.9138(12)
	3.1679(6)		4.0234(11)		
Cs1/K1	3.6116(12)	Cs2/K2	3.7586(7)	Cs2/K2	4.0172(5)
	3.6406(13)		4.4445(9)		4.0340(7)
Cs2/K2	3.8025(10)	Cs3	3.8338(8)	Cs3	4.0386(6)
			4.7015(9)		4.0988(6)
Cs3	3.9981(9)	K3	3.408(12)	K3	3.873(10)
	4.0139(10)				4.129(10)
	4.4842(10)				
K3	3.571(16)	K4	3.6389(5)	K4	3.5491(2)
	4.050(19)				
**Tl4 (8*e*)**		**Cs1/K1 (16*f*)**		**Cs2/K2 (16*f*)**	
Cs2/K2	3.8914(7)	Cs1/K1	4.314(2)	Cs2/K2	4.3256(11)
	4.9328(8)		5.094(2)		
			5.189(2)		
Cs3	4.2599(6)	Cs3	3.9819(16)	Cs3	4.4985(10)
			4.2667(13)		4.4944(10)
			4.4598(14)		4.6835(10)
			4.8158(14)		
K3	3.959(13)	K3	4.376(12)	K3	3.938(17)
			4.415(17)		4.654(13)
			4.419(17)		5.028(11)
			4.745(18)		
K4	3.40595(5)			K4	3.8769(17)
					4.3454(7)
**Cs3 (16*f*)**					
K3	0.64(2)				
K4	4.2920(6)				

6. Supplementary Structure Description

6.1 $Cs_{7.29}K_{5.71}Tl_{13}$

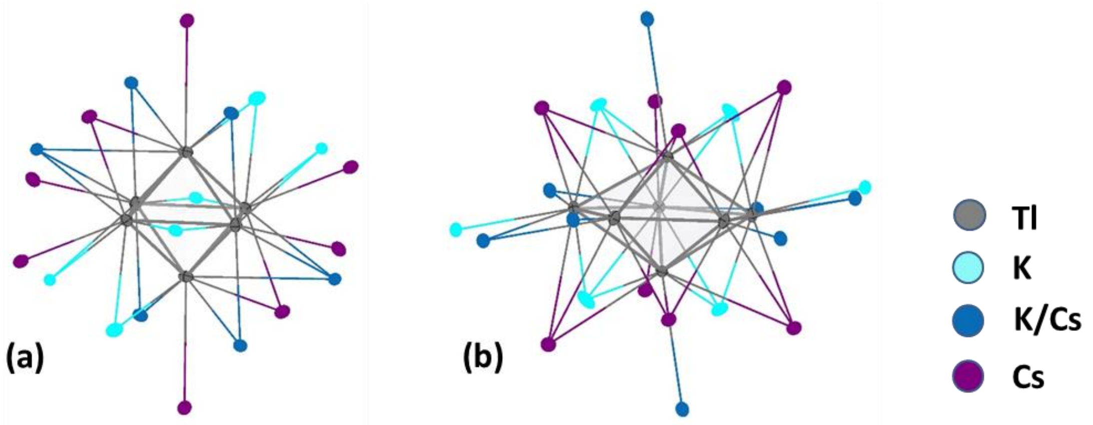

Figure S2: First coordination sphere of the octahedron (a) and the pentagonal bipyramid (b) of the compound $Cs_{7.29}K_{5.71}Tl_{13}$.

The octahedron is surrounded by 20 alkali metal atoms, which exo-coordinate corners, cap edges, or span triangular faces of the cluster unit. The $[Tl_7]^{7-}$ cluster is surrounded by 22 alkali metal atoms, which involve eight cesium atoms (Wyckoff sites 8*f*), six potassium atoms (Wyckoff sites 8*f*/4*a* (Tl5)), and eight mixed occupied positions (Wyckoff sites 8*f*).

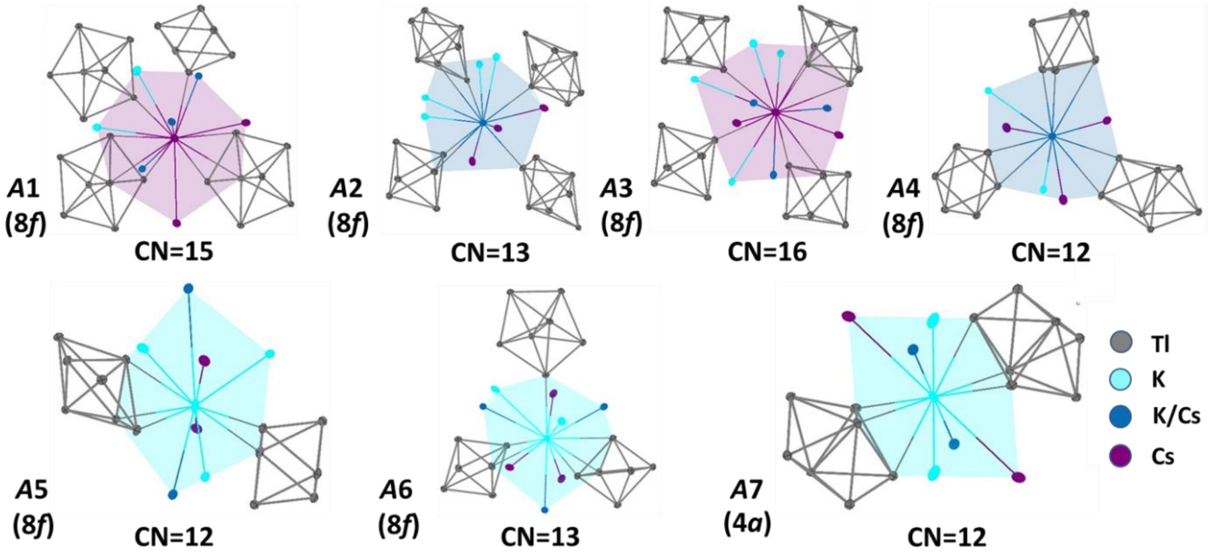

Figure S3: Coordination spheres of the seven crystallographically different alkali metal positions in $Cs_{7.29}K_{5.71}Tl_{13}$.

While cesium-involved alkali metal atoms show coordination numbers (CN) of 12 to 16 and are surrounded by three to four cluster units, the potassium atoms are located between two to three clusters and have CNs of 12 and 13. This emphasizes the need for different alkali metals for the stabilization of the ternary material. The surrounding of one potassium atom (*A*7, Wyckoff position 4*e*) attracts attention, as it is coordinated sandwich like by triangular faces of two pentagonal bipyramids The remaining alkali metal atoms are surrounded by both cluster types.

Table S8: Site occupancy factors (s.o.f) of the mixed occupied alkali metal positions in $Cs_{7.29}K_{5.71}Tl_{13}$.

**s.o.f *A*2 (8*f*)**		**s.o.f. *A*4 (8*f*)**	
Cs2	K1	Cs4	K4
0.864(3)	0.136(3)	0.784(3)	0.216(3)

6.2 $Cs_{3.45}K_{3.55}Tl_7$

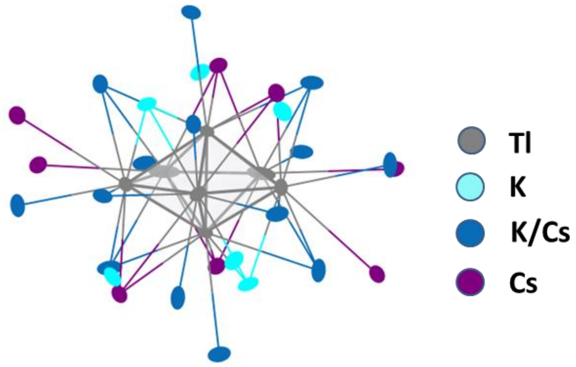

Figure S4: First coordination sphere of the pentagonal bipyramid of the compound $Cs_{3.45}K_{3.55}Tl_7$.

The $[Tl_7]^{7-}$ cluster is again surrounded by 22 alkali metal atoms. While the cluster likewise exhibits ten face capping atoms, here more exo-coordinating alkali metal atoms are present due to elongated distances ($Cs_{7.29}K_{5.71}Tl_{13}$: d(*A*-Tl)≤ 4.5854(5) Å, $Cs_{3.45}K_{3.55}Tl_7$: d(*A*-Tl)≤ 4.9328(8) Å).

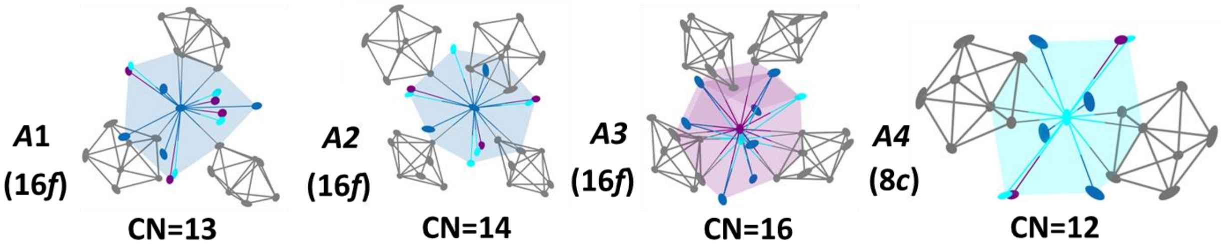

Figure S5: Coordination spheres of the four crystallographically different alkali metal positions in $Cs_{3.45}K_{3.55}Tl_7$. The split positions are represented by one average position in dark blue coloring.

While again cesium involved atoms show a CN of 13 to 16, the potassium atom (*A*4, Wyckoff position 8*c*) is surrounded by 12 next neighbors, which again attracts attention with a very similar surrounding with two face capping Tl_7 clusters. This dense surrounding of the potassium atom by the Tl_7 units is not observed in binary $K_{10}Tl_7$.

Table S9: Site occupancy factors of the mixed occupied alkali metal positions in $Cs_{3.45}K_{3.55}Tl_7$.

**s.o.f. *A*1 (16*f*)**		**s.o.f. *A*2 (16*f*)**		**s.o.f *A*3 (16*f*)**		**s.o.f *A*4 (8*c*)**	
K	0.808(3)	K	0.325(3)	K	0.141(3)	K	1
Cs	0.192(3)	Cs	0.675(3)	Cs	0.859(3)		

7. Cluster Distances in Comparison to Literature-Known Compounds

7.1 Distances of Compressed $[Tl_6]^{6-}$ Octahedra

Table S10: Distance ranges and degree of compression of literature known $[Tl_6]^{6-}$ cluster.

Compound	d(Tl$_{eq}$-Tl$_{eq}$) [Å]	d(Tl$_{eq}$-Tl$_{ap}$) [Å]	d(Tl$_{ap}$-Tl$_{ap}$) [Å]	$d_{ap}/\overline{d_{eq}}$
KTl* [28]	3.3329(6)-<br>3.4585(6)	3.0471(5)-<br>3.0710(5)	3.7898(9)	1.12
CsTl* [29]	3.3932(8)-<br>3.4130(7)	3.0216(5)-<br>3.0772(5)	3.7477(10)	1.10
$Cs_{7.29}K_{5.71}Tl_{13}$	3.3418(3)-<br>3.3687(3)	3.0143(3)-<br>3.1203(3)	3.8819(4)	1.16
$Rb_{10}Tl_6O_2$ [30]	3.415(2)-<br>3.654(2)	3.014(1)-<br>3.090(1)	3.574(2)	1.01
$K_{10}Tl_6O_2$ [30]	3.390(1)-<br>3.704(1)	3.010(1)-<br>3.094(1)	3.578(1)	1.01
$Cs_{10}Tl_6SiO_4$ [31]	3.388(2)-<br>3.394(2)	3.001(2)-<br>3.116(2)	3.772(7)	1.11
	3.407(2)-<br>3.414(2)	3.035(2)-<br>3.117(2)	3.800(7)	1.11
$Cs_{10}Tl_6SnO_3$ [31]	3.394(1)-<br>3.474(1)	3.033(1)-<br>3.105(1)	3.371(1)	0.98

7.2 Distances of Pentagonal Bipyramids $[Tl_7]^{7-}$

Table S11: Distance ranges and degree of compression of literature known $[Tl_7]^{7-}$ cluster.

Compound	d(Tl$_{eq}$-Tl$_{eq}$) [Å]	d(Tl$_{eq}$-Tl$_{ap}$) [Å]	d(Tl$_{ap}$-Tl$_{ap}$) [Å]	$d_{ap}/\overline{d_{eq}}$
$K_{10}Tl_7$ [32]	3.183(1)-<br>3.247(1)	3.185(1)-<br>3.301(1)	3.4622(9)	1.08
$Na_9K_{16}Tl_{\sim 25}$ [33]	3.162(2)-<br>3.388(1)	3.180(2)-<br>3.352(2)	3.354(2)	1.02
$Na_{12}K_{38}Tl_{48}Au_2$ [34]	3.207(4)-<br>3.389(3)	3.267(2)-<br>3.293(3)	3.394(4)	1.03
$Cs_{7.29}K_{5.71}Tl_{13}$	3.1194(3)-<br>3.2406(4)	3.1323(3)-<br>3.2421(3)	3.3692(4)	1.06
$Cs_{3.45}K_{3.55}Tl_7$	3.1526(6)-<br>3.2041(10)	3.1485(6)-<br>3.2164(5)	3.3635(3)	1.06

8. Experimental Solid State Approaches

Table S12: Approaches with potassium and cesium in the A:Tl ratio 1:1.

Sample	Temperature Program	m(Tl) /g	m(K) /g	m(Cs) /g	Phases	Characterized by
Cs_2KTl_3	1	0.7365	0.0470	0.2977	$Cs_{3.45}K_{3.55}Tl_7$<br>$Cs_{7.29}K_{5.71}Tl_{13}$<br>$Cs_{8-x}K_xTl_{11}$	SCXRD, PXRD
CsK_2Tl_3	1	0.7163	0.0914	0.1553	$Cs_{3.45}K_{3.55}Tl_7$<br>KTl<br>$Cs_{8-x}K_xTl_{11}$ (a)	SCXRD, EDS
$KCsTl_2$	2	0.7593	0.0726	0.2469	$Cs_{3.45}K_{3.55}Tl_7$<br>$Cs_{8-x}K_xTl_{11}$ (b)	PXRD
$KCsTl_2$	1	0.7320	0.0700	0.2380	$Cs_{3.45}K_{3.55}Tl_7$<br>$Cs_{7.29}K_{5.71}Tl_{13}$	SCXRD, PXRD
$Cs_{3.45}K_{3.55}Tl_7$	1	0.7020	0.0683	0.2243	$Cs_{3.45}K_{3.55}Tl_7$<br>$Cs_{7.29}K_{5.71}Tl_{13}$	PXRD
$Cs_{3.45}K_{3.55}Tl_7$	2	0.7292	0.0707	0.2337	$Cs_{3.45}K_{3.55}Tl_7$<br>$Cs_{8-x}K_xTl_{11}$	PXRD
$Cs_{7.29}K_{5.71}Tl_{13}$	2	0.7790	0.0653	0.2845	$Cs_{3.45}K_{3.55}Tl_7$<br>$Cs_{8-x}K_xTl_{11}$	PXRD
$Cs_{7.65}K_{6.0}Tl_{13}$	1	0.6323	0.0558	0.2420	$Cs_{3.45}K_{3.55}Tl_7$<br>$Cs_{7.29}K_{5.71}Tl_{13}$<br>$Cs_{8-x}K_xTl_{11}$	PXRD
$Cs_{7.65}K_{6.0}Tl_{13}$	2	0.6862	0.0606	0.2626	$Cs_{3.45}K_{3.55}Tl_7$<br>$Cs_{8-x}K_xTl_{11}$	PXRD
$Cs_4K_3Tl_7$	1	0.7322	0.0600	0.2720	$Cs_{3.45}K_{3.55}Tl_7$<br>$Cs_{7.29}K_{5.71}Tl_{13}$<br>$Cs_{8-x}K_xTl_{11}$ (c)	SCXRD, PXRD, EDS

(a) Cell parameter (RT): a=b=10.28121 Å, c=52.0477 Å. Vegard's law[35] was used to determine the composition $K_{3.826}Cs_{4.174}Tl_{11}$.

(b) Cell parameter (RT): a=b=10.27812 Å, c=51.35844 Å. Vegard's law[35] was used to determine the composition $K_{3.87}Cs_{4.13}Tl_{11}$.

(c) Cell parameter (RT): a=b=10.28335 Å, c=52.109566 Å. Vegard's law[35] was used to determine the composition $K_{3.79}Cs_{4.21}Tl_{11}$.

Temperature programs:

1)

RT —100 °/h→ 773.15 K —48 h→ 773.15 K —5 °/h→ RT

2)

RT —100 °/h→ 773.15 K —48 h→ 773.15 K —quenching in water→ RT

8.1 Powder Diffraction Pattern of the Approach Cs_2KTl_3

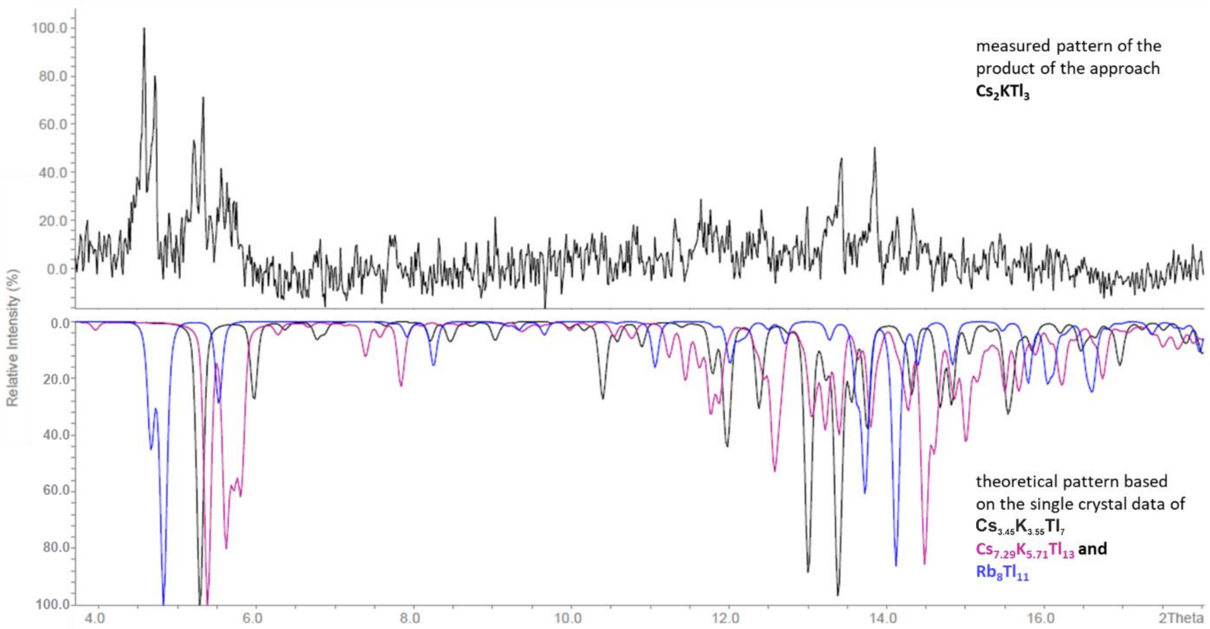

Figure S6: Powder diffraction pattern of the approach Cs_2KTl_3 (black, up) with the theoretical pattern of $Cs_{3.45}K_{3.55}Tl_7$ (down, black) , $Cs_{7.29}K_{5.71}Tl_{13}$ (down, pink) and Rb_8Tl_{11} (down, blue) as an average pattern between K_8Tl_{11} and Cs_8Tl_{11}.There are additional reflections, which yet cannot yet be assigned to any literature known phase. Further investigations are in progress. Indexation using JANA2006 was not possible due to weak data.

8.2 Powder Diffraction Pattern of the Approach CsK_2Tl_3

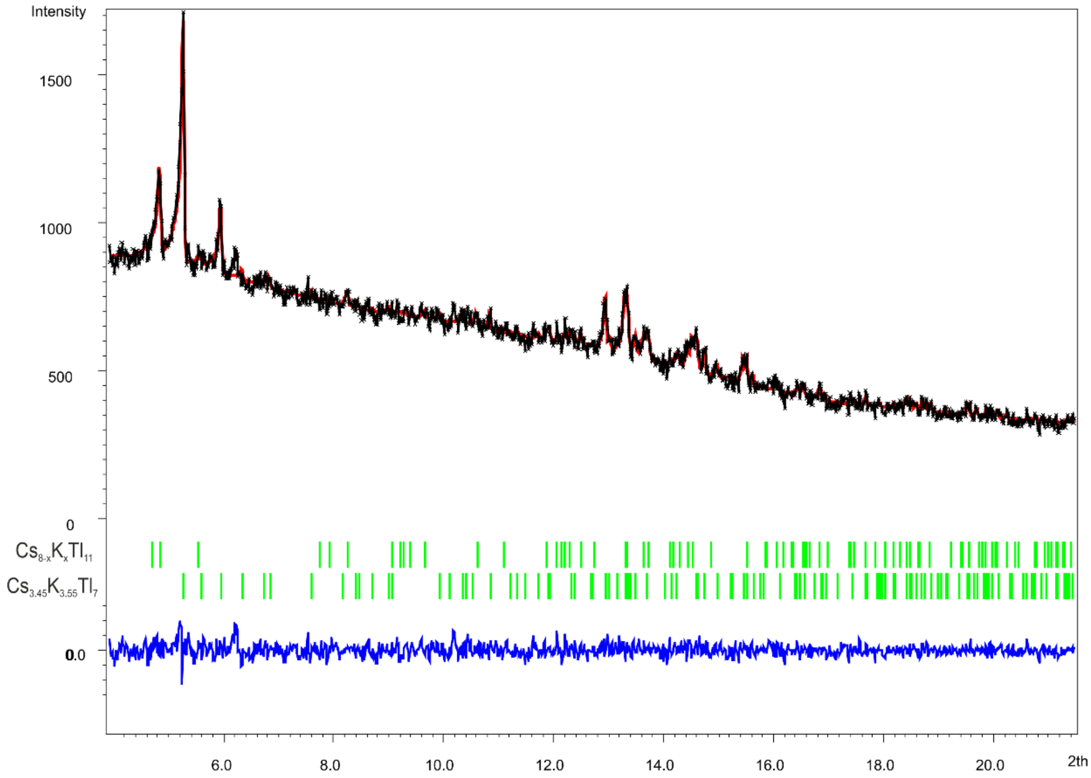

Figure S7: Measured powder diffraction pattern of the product of the sample CsK_2Tl_3 (black). The refinement was carried out with the LeBail algorithm in JANA2006. The vertical bars (green) underneath the powder pattern show the calculated reflection positions. The curve at the bottom (blue) represents the difference plot. GOF=0.76, R_p=2.81, R_{wp}=3.70.

8.3 Powder Diffraction Pattern of the Approach $CsKTl_2$ (Temp. 1)

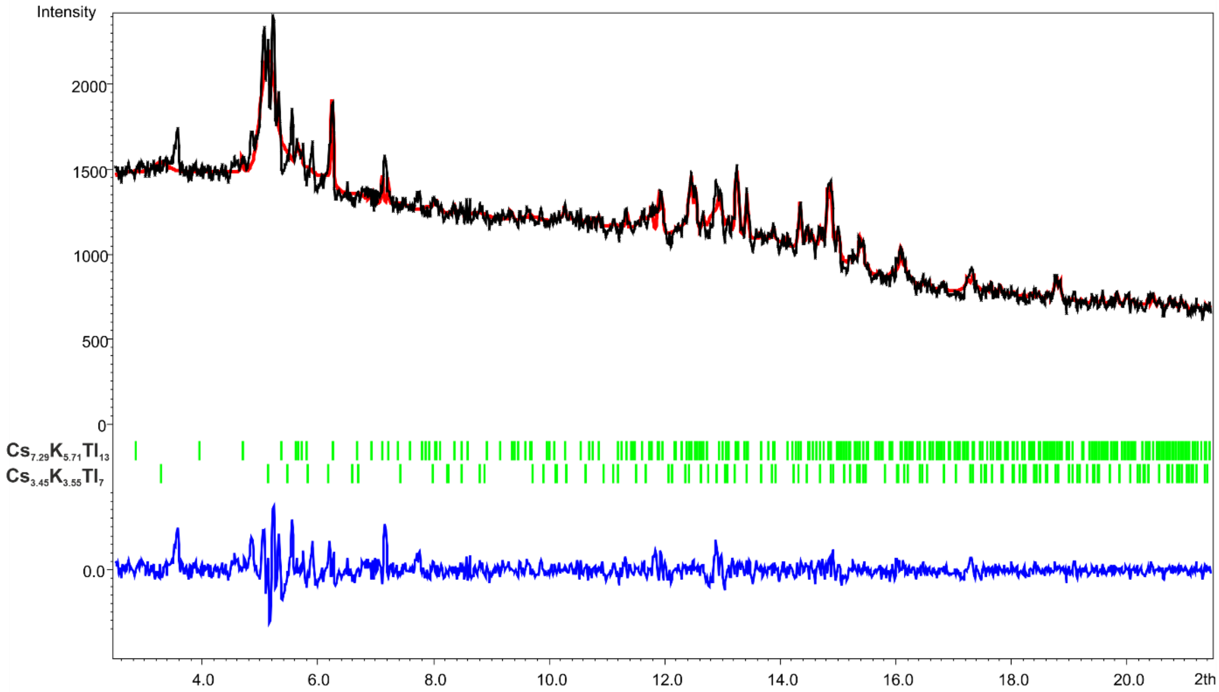

Figure S8: Measured powder diffraction pattern of the product of the sample $CsKTl_2$ (black). The refinement was carried out with the LeBail algorithm in JANA2006. The vertical bars (green) underneath the powder pattern show the calculated reflection positions. The curve at the bottom (blue) represents the difference plot. GOF=1.00, R_p=2.57, R_{wp}=3.56. The two title compounds can be indexed, but the additional reflections cannot yet be assigned. It also cannot be ruled out that $Cs_{8-x}K_xTl_{11}$ is also present (unrefined reflection at 2th=4.8°).

8.4 Powder Diffraction Pattern of the Approach $CsKTl_2$ (Temp. 2)

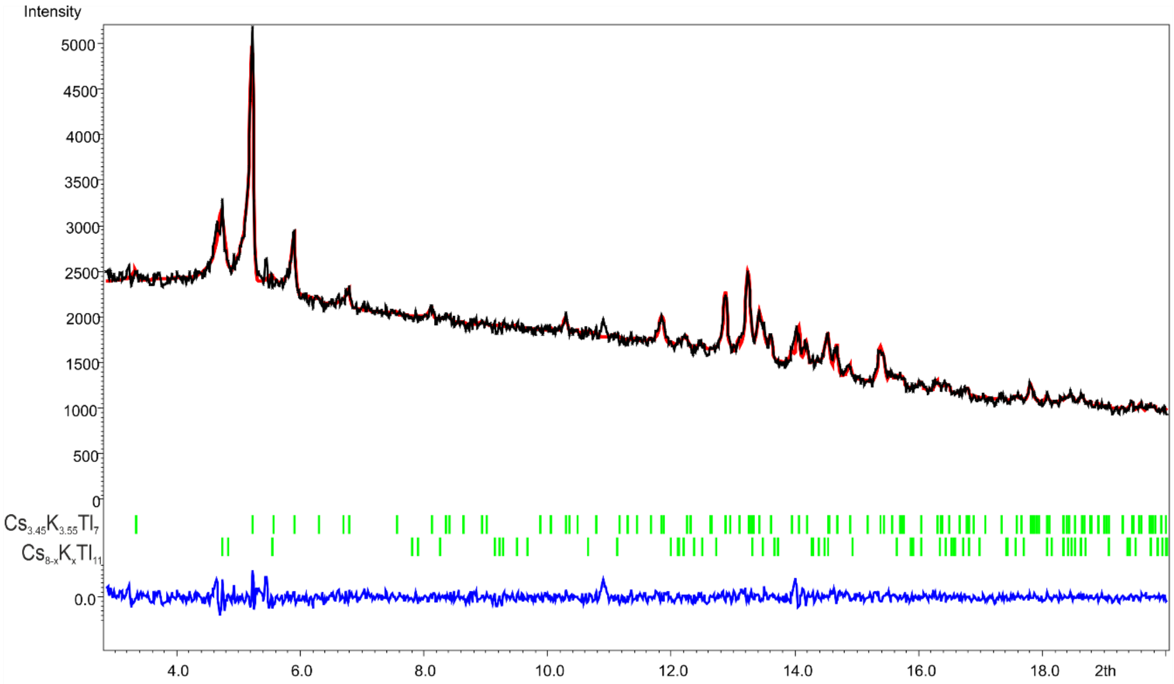

Figure S9: Measured powder diffraction pattern of the product of the sample $CsKTl_2$ (black). The refinement was carried out with the LeBail algorithm in JANA2006. The vertical bars (green) underneath the powder pattern show the calculated reflection positions. The curve at the bottom (blue) represents the difference plot. GOF=0.84, R_p=1.85, R_{wp}=2.45.

8.5 Powder Diffraction Pattern of the Approach $Cs_{3.45}K_{3.55}Tl_7$ (Temp. 1)

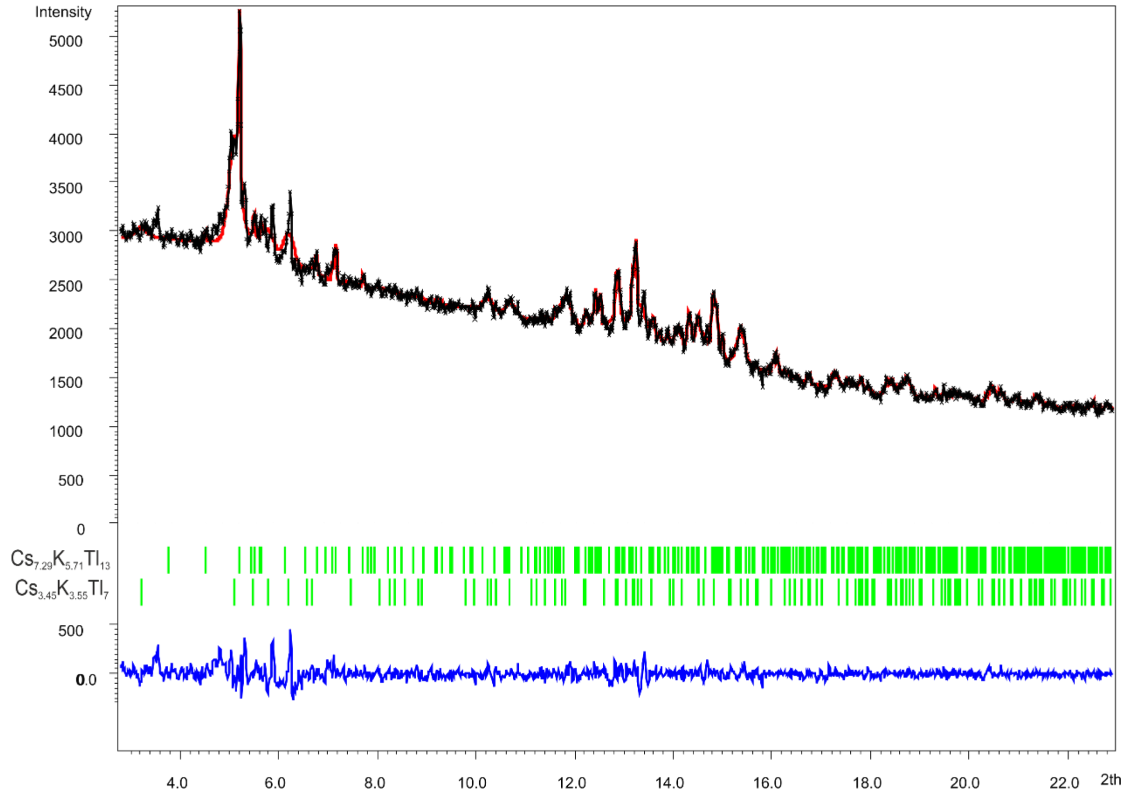

Figure S10: Measured powder diffraction pattern of the product of the sample $Cs_{3.45}K_{3.55}Tl_7$ (black). The refinement was carried out with the LeBail algorithm. The calculated reflection positions are shown by the vertical bars (green) underneath the powder pattern. The curve at the bottom (blue) represents the difference plot. GOF=0.95 , R_p=1.72, R_{wp}=2.42. It cannot be ruled out that $Cs_{8-x}K_xTl_{11}$ is also present.

8.6 Powder Diffraction Pattern of the Approach $Cs_{3.45}K_{3.55}Tl_7$ (Temp. 2)

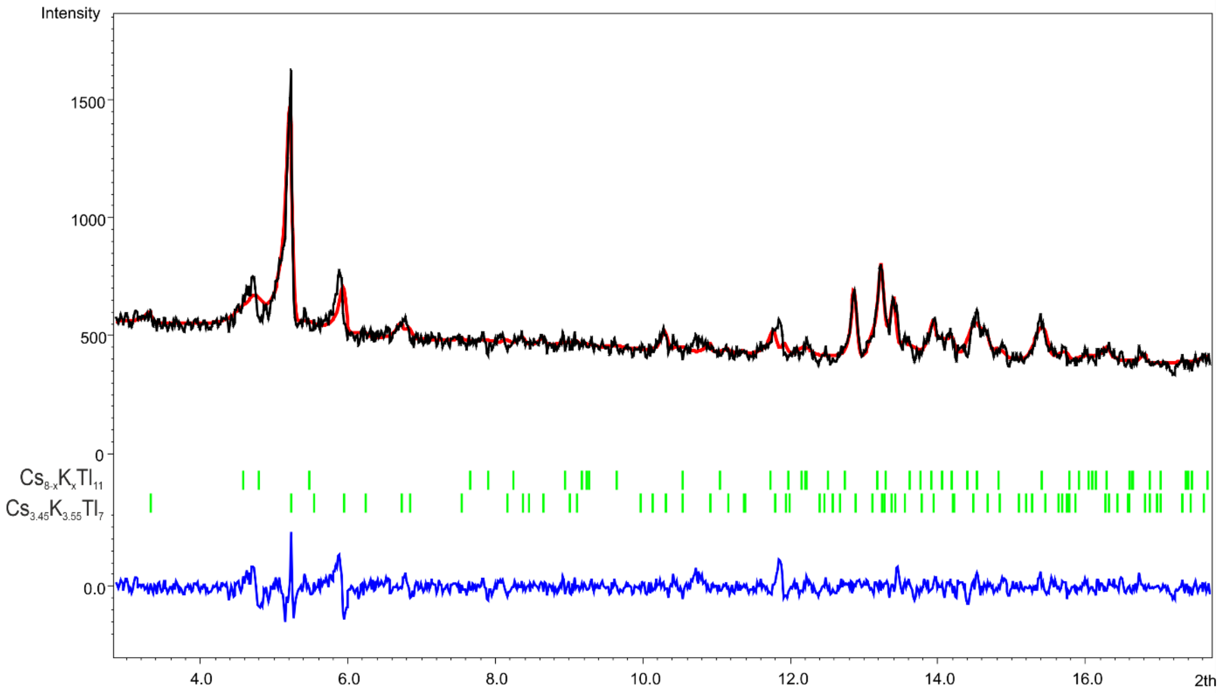

Figure S11: Measured powder diffraction pattern of the product of the sample $Cs_{3.45}K_{3.55}Tl_7$ (black). The refinement was carried out with the LeBail algorithm. The calculated reflection positions are shown by the vertical bars (green) underneath the powder pattern. The curve at the bottom (blue) represents the difference plot. GOF=0.95 , R_p=3.54, R_{wp}=4.80.

8.7 Powder Diffraction Pattern of the Approach $Cs_{7.29}K_{5.71}Tl_{13}$

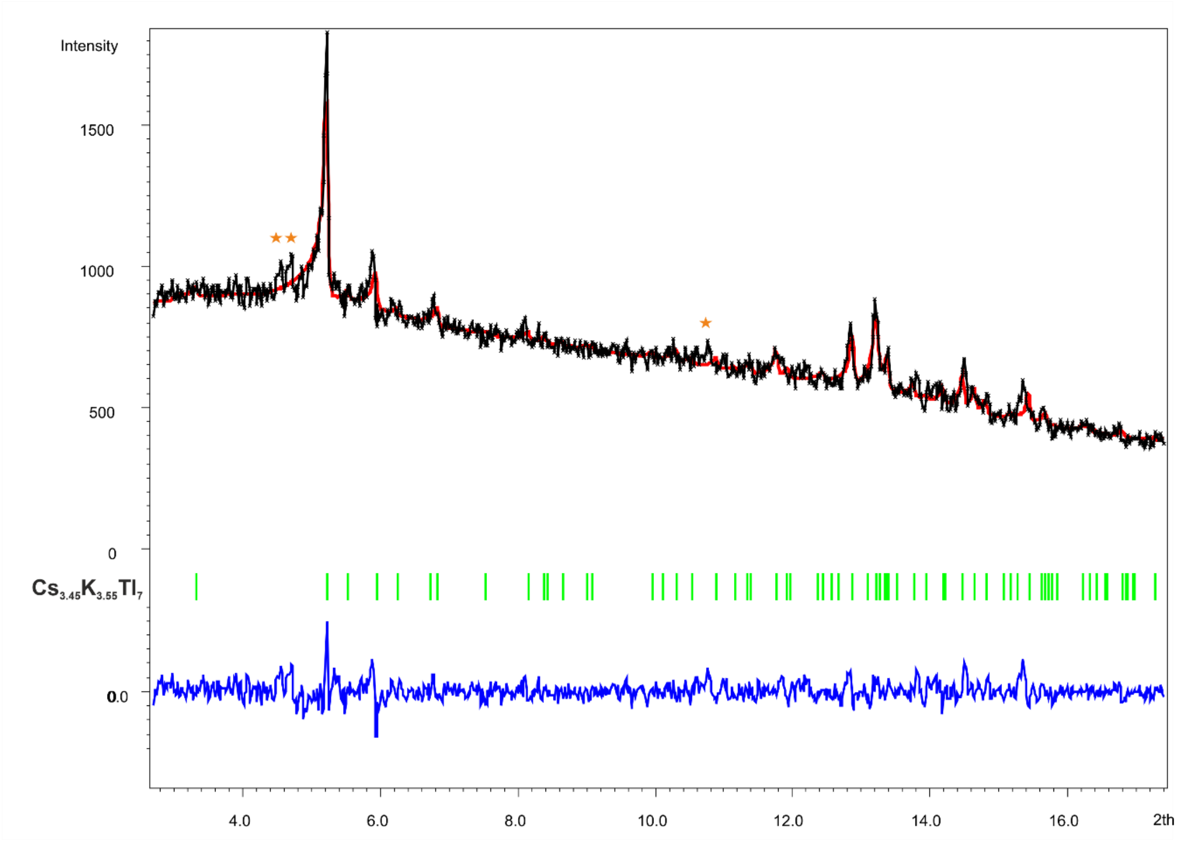

Figure S12: Measured powder diffraction pattern of the product of the sample $Cs_{7.29}K_{5.71}Tl_{13}$ (black). The refinement was carried out with the LeBail algorithm. The calculated reflection positions are shown by the vertical bars (green) underneath the powder pattern. The curve at the bottom (blue) represents the difference plot. GOF=0.91 , R_p=3.41, R_{wp}=4.47. The reflections marked with yellow stars indicate a $Cs_{8-x}K_xTl_{11}$ phase, but due to the low intensity a refinement with the LeBail algorithm was not successful.

8.8 Powder Diffraction Pattern of the Approach $Cs_{7.65}K_6Tl_{13}$ (Temp. 1)

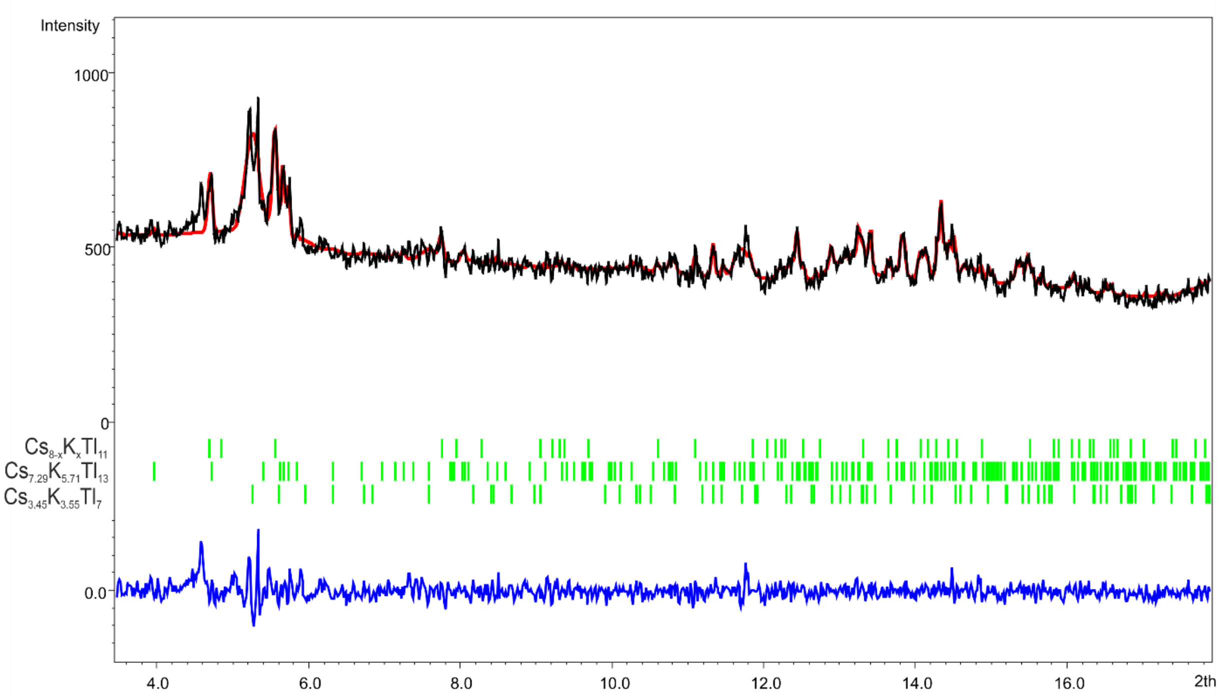

Figure S13: Measured powder diffraction pattern of the product of the sample $Cs_{7.65}K_6Tl_{13}$ (black). The refinement was carried out with the LeBail algorithm. The calculated reflection positions are shown by the vertical bars (green) underneath the powder pattern. The curve at the bottom (blue) represents the difference plot. GOF=0.76 , R_p=2.88, R_{wp}=4.01.

8.9 Powder Diffraction Pattern of the Approach $Cs_{7.65}K_6Tl_{13}$ (Temp. 2)

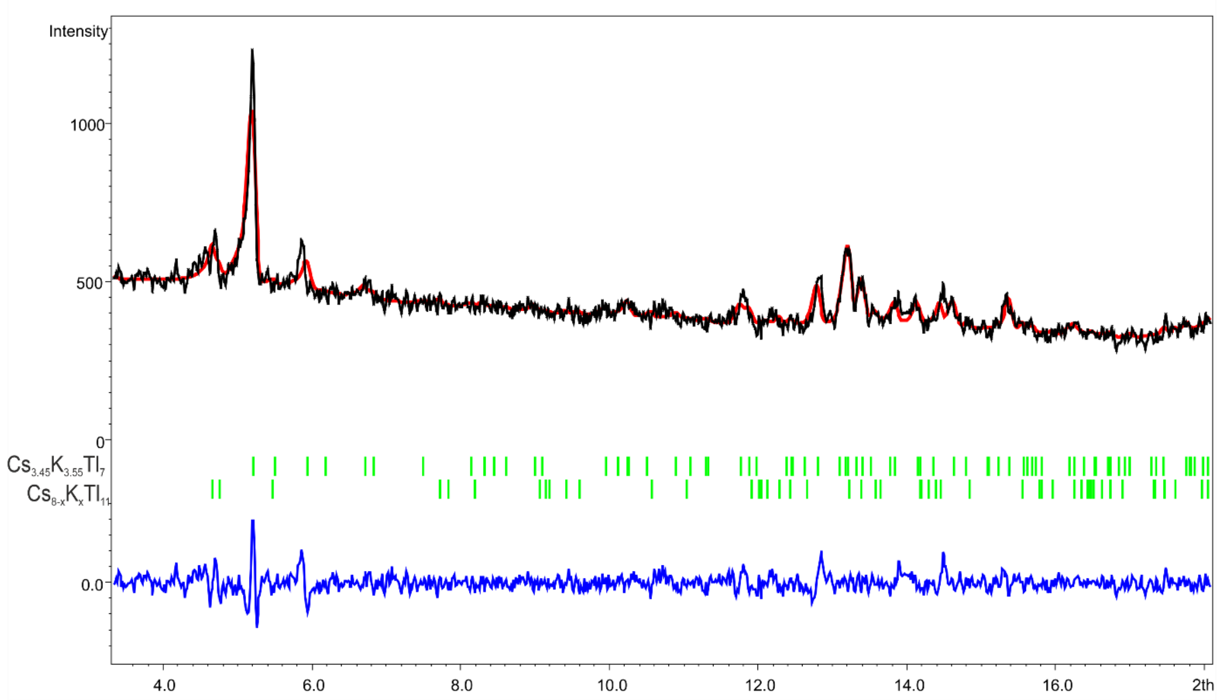

Figure S14: Measured powder diffraction pattern of the product of the sample $Cs_{7.65}K_6Tl_{13}$ (black). The refinement was carried out with the LeBail algorithm. The calculated reflection positions are shown by the vertical bars (green) underneath the powder pattern. The curve at the bottom (blue) represents the difference plot. GOF=0.93, R_p=3.78, R_{wp}=5.07.

8.10 Powder Diffraction Pattern of the Approach $Cs_4K_3Tl_7$

The determination of the ratio of the three phases $Cs_{3.45}K_{3.55}Tl_7$, $Cs_{7.29}K_{5.71}Tl_{13}$, and $K_{3.79}Cs_{4.21}Tl_{11}$ is not possible due to the poor quality of the powder diffractogram. The presence of amorphous products cannot be ruled out either.

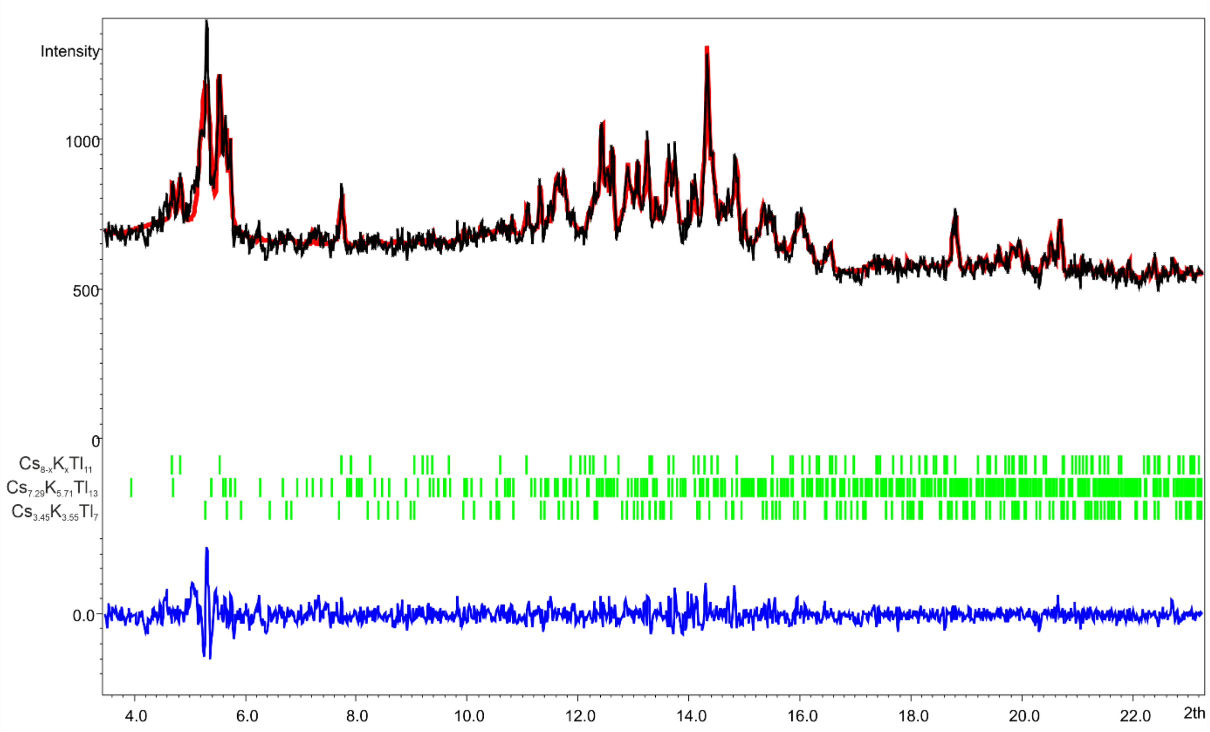

Figure S15: Measured powder diffraction pattern of the product of the sample $Cs_4K_3Tl_7$ (black). The refinement was carried out with the LeBail algorithm. The calculated reflection positions are shown by the vertical bars (green) underneath the powder pattern. The curve at the bottom (blue) represents the difference plot. GOF=0.79 , R_p=2.22, R_{wp}=3.18.

9. Packing Order of the $[Tl_7]^{7-}$ Cluster in $Cs_{3.45}K_{3.55}Tl_7$

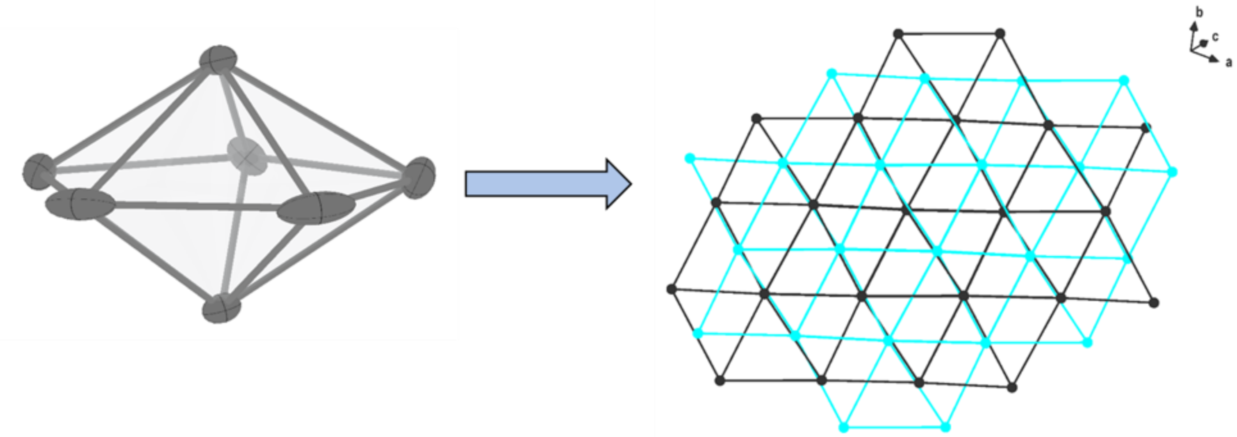

Figure S16: The Tl moiety in $Cs_{3.45}K_{3.55}Tl_7$ is represented by the $[Tl_7]^{7-}$ pentagonal bipyramids, which are arranged as hexagonal layers arranged in an AB stacking sequence (on the right in black and blue). This layer arrangement can be traced back to a distorted α-uranium type.

10. Band Structure

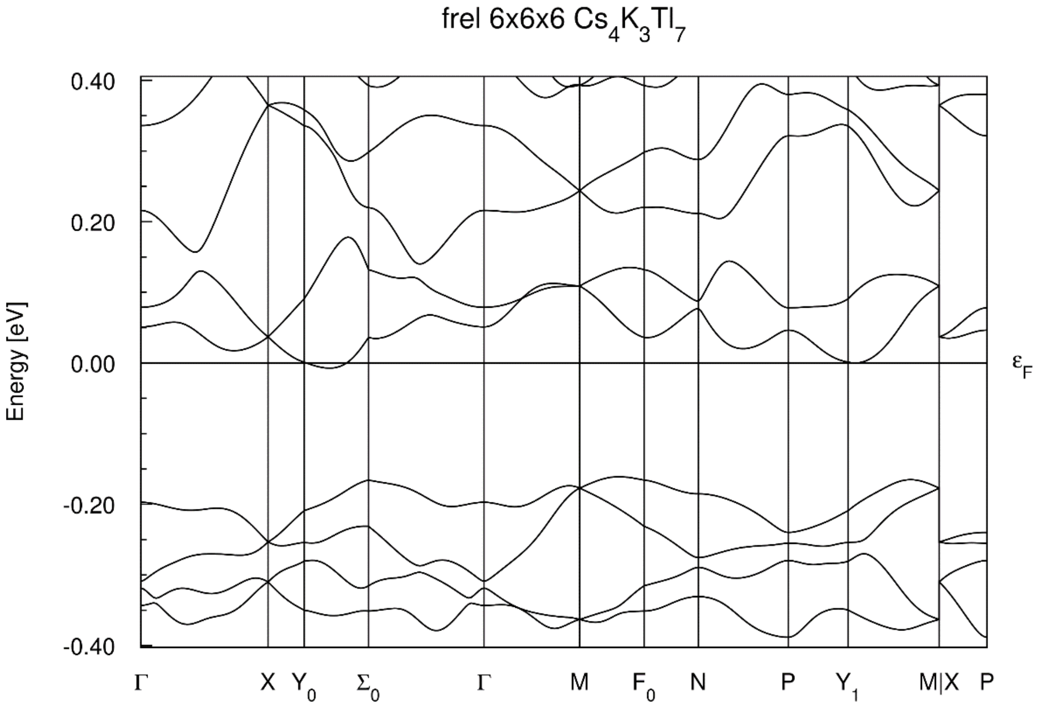

Figure S17: Excerpt of the band structure of $Cs_4K_3Tl_7$.

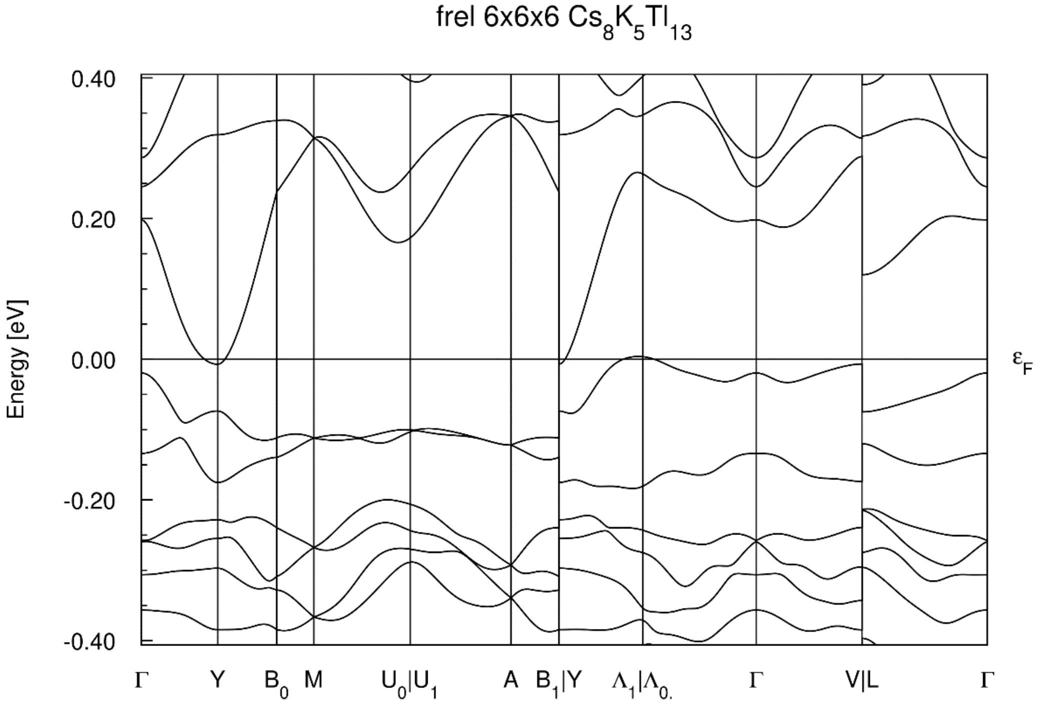

Figure S18: Excerpt of the band structure of $Cs_8K_5Tl_{13}$.

11. Dissolution Experiments in Liquid Ammonia

After preparing the vessel in the glove box, dried liquid ammonia was condensed onto the Cs_2KTl_3 sample. The color of the solution changed to dark blue, which indicated solvated electrons. The solution was stored for five months at 233 K. The liquid ammonia of the then colorless solution with a grey precipitate was evaporated and a grey and white residue was left.

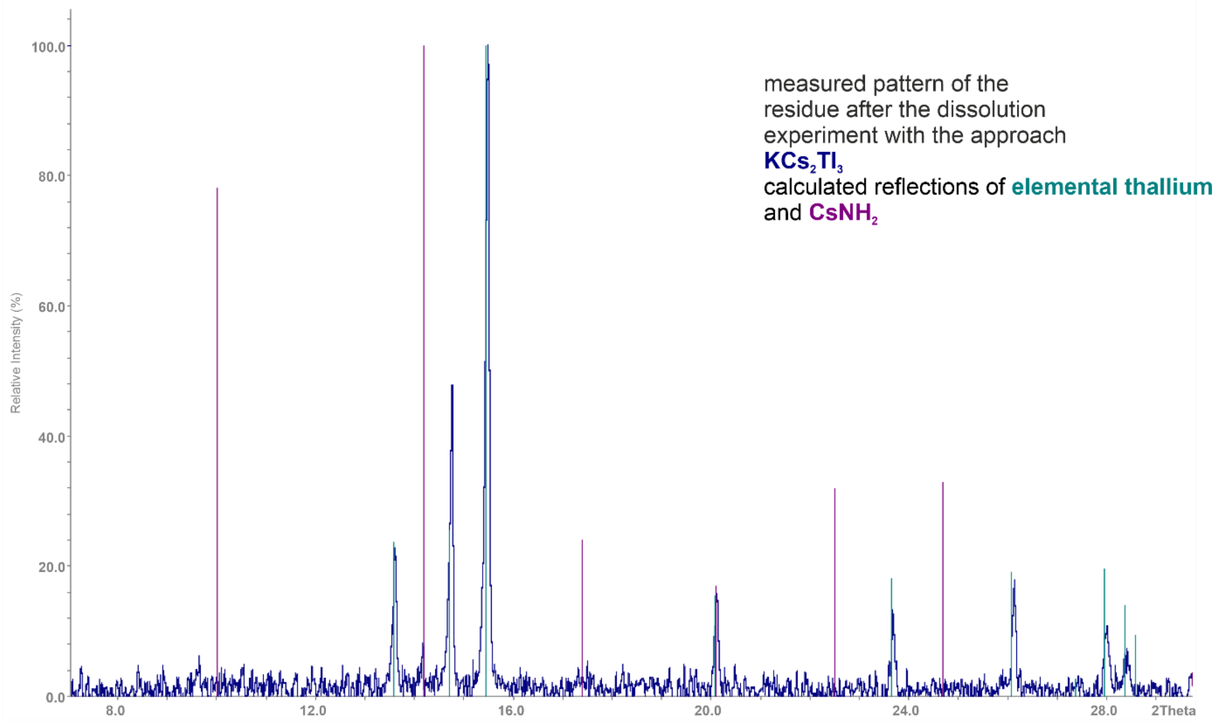

Figure S19: Measured powder diffraction pattern of the residue after the dissolution experiment in liquid ammonia of the approach Cs_2KTl_3 Only the strongest reflection of $CsNH_2$ is observed.

In this measured powder diffraction pattern of the residue after the dissolution experiment of the approach Cs_2KTl_3 elemental thallium could be indexed very well. The strongest not-indexed reflection at 14.2° indicates the presence of $CsNH_2$.

12. SEM/EDS Measurements

12.1 Measurements of the Approach K_2CsTl_3

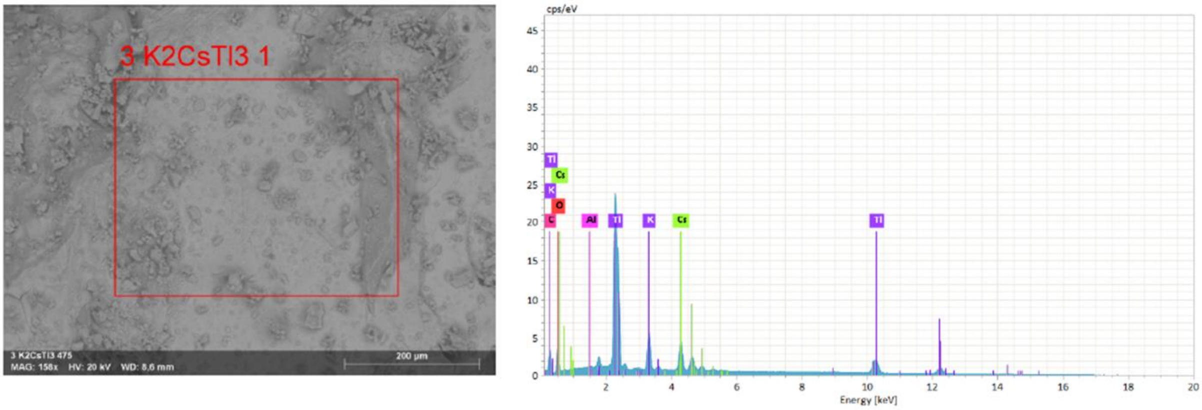

Figure S20:EDS spectrum (right) and the SEM picture of the crystal of the approach K_2CsTl_3 with the measured area marked with the red rectangle (left).

Table S13: Analysis of the EDS spectrum.

Element	At. No.	Netto	Mass [%]	Mass Norm. [%]	Atom [%]	Abs. error [%] (1σ)	Rel. Error [%] (1σ)
Tl	81	37342	64.15	70.50	48.49	1.94	3.03
K	19	37907	7.28	8.00	28.78	0.25	3.43
Cs	55	58386	19.56	21.49	22.73	0.57	2.93
C	6	7755	0	0	0	0	0
O	8	17129	0	0	0	0	0
Al	13	1379	0	0	0	0	0

The EDS measurement of a crystal from the sample K_2CsTl_3 (see Figure S13 and Table S13) revealed, taking the error into account, that the sample matches the composition $Cs_{3.45}K_{3.55}Tl_7$ of one of the title compounds quite well (atomic percentage calculated/measured: 50/48.49% (Tl), 24.7/22.73% (Cs), 25.3/28.78% (K)). The slight amounts of carbon, oxygen, and aluminum are possibly from the adhesive, the sample carrier, and minimal contamination due to possible leaks during the transport of the samples.

A different crystallite, which was analyzed with SEM/EDS did not contain cesium (see Figure S14). A closer look at the spectrum and the corresponding evaluation yields an approximate composition of

53.83% of thallium and 46.17% of potassium (see Table S14) which can be assigned KTl. Single crystals of KTl were also isolated from this approach.

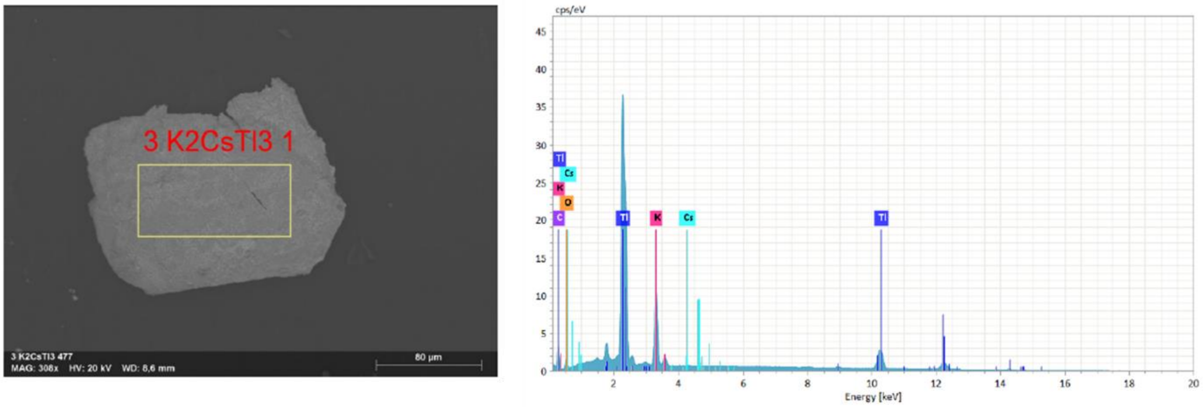

Figure S21: EDS spectrum (right) and the SEM picture of another crystal of the approach K_2CsTl_3 with the measured area marked with the red rectangle (left).

Table S14: Analysis of the EDS spectrum.

**Element**	**At. No.**	**Netto**	**Mass [%]**	**Mass Norm. [%]**	**Atom [%]**	**Abs. error [%] (1σ)**	**Rel. Error [%] (1σ)**
Tl	81	69280	71.73	85.90	53.83	2.15	3.00
K	19	101930	11.77	14.10	46.17	0.38	3.26
Cs	55	46	0	0	0	0	0
C	6	8809	0	0	0	0	0
O	8	10550	0	0	0	0	0

12.2 Measurements on the Approach $Cs_4K_3Tl_7$

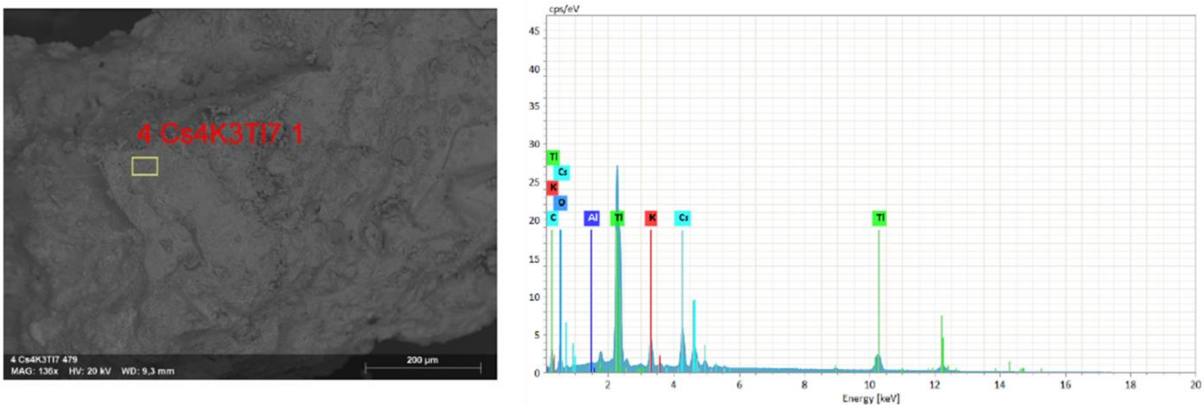

Figure S22: EDS spectrum (right) and the SEM picture of a crystal of the approach $Cs_4K_3Tl_7$ with the measured area marked with the yellow rectangle (left).

Table S15: Analysis of the EDS spectrum.

Element	At. No.	Netto	Mass [%]	Mass Norm. [%]	Atom [%]	Abs. error [%] (1σ)	Rel. Error [%] (1σ)
Tl	81	29622	64.90	70.54	52.72	1.98	3.05
K	19	19730	4.48	4.87	19.01	0.17	3.70
Cs	55	57078	22.63	24.59	28.27	0.66	2.91
C	6	4975	0	0	0	0	0
O	8	12893	0	0	0	0	0
Al	13	845	0	0	0	0	0

The EDS measurement of a crystal from the sample $Cs_4K_3Tl_7$ (see Figure S15 and Table S15) showed, taking the error into account, that the sample matches the composition $Cs_{7.29}K_{5.71}Tl_{13}$ quite well (atomic percentage calculated/measured: 50/52.72% (Tl), 28.04/28.27% (Cs), 21.96/19.01% (K)).

13. Literature

(1) Hackspill, L. Sur une nouvelle préparation du rubidium et du caesium. *Comptes Rendus Hebdomadaires de Seances de l'Academie des Sciences* **1905**.
(2) *CrysAlisPro*; Rigaku Oxford Diffraction/Agilent Technologies UK Ltd: Yarnton, UK, 2020.
(3) Dolomanov, O. V.; Bourhis, L. J.; Gildea, R. J.; Howard, J. A. K.; Puschmann, H. OLEX^2: A complete structure solution, refinement and analysis program *J. Appl. Crystallogr.* **2009**, *42*, 339-341. DOI: 10.1107/S0021889808042726.
(4) Sheldrick, G. M. A short history of SHELX. *Acta Cryst. A* **2008**, *64* (1), 112-122.
(5) Sheldrick, G. M. SHELXT - Integrated space-group and crystal-structure determination. *Acta Crystallogr A* **2015**, *71*, 3-8, Article. DOI: 10.1107/s2053273314026370.
(6) Sheldrick, G. M. Crystal structure refinement with SHELXL. *Acta Crystallogr C* **2015**, *71*, 3-8. DOI: 10.1107/s2053229614024218.
(7) Bourhis, L. J.; Dolomanov, O. V.; Gildea, R. J.; Howard, J. A. K.; Puschmann, H. The anatomy of a comprehensive constrained, restrained refinement program for the modern computing environment-*Olex2* dissected. *Acta Crystallogr. A* **2015**, *71*, 59-75. DOI: 10.1107/s2053273314022207.
(8) Herbst-Irmer, R.; Henn, J.; Holstein, J. J.; Hübschle, C. B.; Dittrich, B.; Stern, D.; Kratzert, D.; Stalke, D. Anharmonic Motion in Experimental Charge Density Investigations. *J. Phys. Chem. A* **2013**, *117* (3), 633-641. DOI: 10.1021/jp309985e.
(9) Kuhs, W. F. The anharmonic temperature factor in crystallographic structure-analysis. *Aus. J. Phys.* **1988**, *41* (3), 369-382. DOI: 10.1071/ph880369.
(10) *Diamond*; Crystal Impact GbR: Bonn, Germany, 2019.
(11) *STOE WinXPOW*; STOE & Cie GmbH: Darmstadt, 2016.
(12) Petricek, V.; Dusek, M.; Palatinus, L. Crystallographic Computing System JANA2006: general features *Z. Kristallogr.*, **2014**; *229*, 345-352.
(13) Koepernik, K.; Eschrig, H. Full-potential nonorthogonal local-orbital minimum-basis band-structure scheme. *Phys. Rev. B* **1999**, *59* (3), 1743-1757. DOI: 10.1103/PhysRevB.59.1743.
(14) Opahle, I.; Koepernik, K.; Eschrig, H. Full-potential band-structure calculation of iron pyrite. *Phys. Rev. B* **1999**, *60* (20), 14035-14041. DOI: 10.1103/PhysRevB.60.14035.
(15) Eschrig, H.; Koepernik, K.; Chaplygin, I. Density functional application to strongly correlated electron systems. *J. Solid State Chem.* **2003**, *176* (2), 482-495. DOI: 10.1016/s0022-4596(03)00274-3.
(16) Lejaeghere, K.; Bihlmayer, G.; Bjorkman, T.; Blaha, P.; Blugel, S.; Blum, V.; Caliste, D.; Castelli, I. E.; Clark, S. J.; Dal Corso, A.; et al. Reproducibility in density functional theory calculations of solids. *Science* **2016**, *351* (6280), 7. DOI: 10.1126/science.aad3000.
(17) Perdew, J. P.; Burke, K.; Ernzerhof, M. Generalized gradient approximation made simple. *Physical Review Letters* **1996**, *77* (18), 3865-3868. DOI: 10.1103/PhysRevLett.78.1396.
(18) *Origin(Pro)*; OriginLab Corporation: Northampton, MA, USA, 2022. (accessed.
(19) Parthe, E.; Gelato, L. M. The standardization of inorganic crystal-structure data. *Acta Crystallogr. A* **1984**, *40* (MAY), 169-183. DOI: 10.1107/s0108767384000416.
(20) Gelato, L. M.; Parthe, E. STRUCTURE TIDY - a computer-program to standardize crystal-structure data. *J. Appl. Crystallogr.* **1987**, *20*, 139-143. DOI: 10.1107/s0021889887086965.
(21) Hu, S. Z.; Parthé, E. Inorganic crystal structure data to be presented in a form more useful for further studies. *Chin. J. Struct. Chem.* **2004**, *23* (10), 1150-1160.
(22) Spek, A. L. Single-crystal structure validation with the program *PLATON*. *J. Appl. Crystallogr.* **2003**, *36*, 7-13. DOI: 10.1107/s0021889802022112.
(23) Spek, A. L. Structure validation in chemical crystallography. *Acta Crystallogr. D* **2009**, *65*, 148-155. DOI: 10.1107/s090744490804362x.
(24) Spek, A. L. What makes a crystal structure report valid? *Inorg. Chim. Acta* **2018**, *470*, 232-237. DOI: 10.1016/j.ica.2017.04.036.

(25) Aroyo, M. I.; Kirov, A.; Capillas, C.; Perez-Mato, J. M.; Wondratschek, H. Bilbao crystallographic server. II. Representations of crystallographic point groups and space groups. *Acta Crystallogr. A* **2006**, *62*, 115-128. DOI: 10.1107/s0108767305040286.
(26) Aroyo, M. I.; Perez-Mato, J. M.; Capillas, C.; Kroumova, E.; Ivantchev, S.; Madariaga, G.; Kirov, A.; Wondratschek, H. Bilbao crystallographic server: I. Databases and crystallographic computing programs. *Z. Kristallogr.* **2006**, *221* (1), 15-27. DOI: 10.1524/zkri.2006.221.1.15.
(27) Aroyo, M. I.; Perez-Mato, J. M.; Orobengoa, D.; Tasci, E.; de la Flor, G.; Kirov, A. Crystallography online: Bilbao Crystallographic Server. *Bulg. Chem. Commun.* **2011**, *43* (2), 183-197.
(28) Dong, Z. C.; Corbett, J. D. Synthesis, Structure, and Bonding of the Novel Cluster Compound KTl with Isolated Tl_6^{6-} Ions. *J. Am. Chem. Soc.* **1993**, *115* (24), 11299-11303. DOI: 10.1021/ja00077a031.
(29) Dong, Z. C.; Corbett, J. D. CsTl: A new example of tetragonally compressed Tl_6^{6-} octahedra. Electronic effects and packing requirements in the diverse structures of ATl (A=Li, Na, K, Cs). *Inorg. Chem.* **1996**, *35* (8), 2301-2306. DOI: 10.1021/ic951265v.
(30) Karpov, A.; Jansen, M. $A_{10}Tl_6O_2$ (A = K, Rb) cluster compounds combining structural features of thallium cluster anions and of alkali metal sub-oxides. *Chem. Commun.* **2006**, (16), 1706-1708. DOI: 10.1039/b601802e.
(31) Saltykov, V.; Nuss, J.; Jansen, M. $Cs_{10}Tl_6SiO_4$, $Cs_{10}Tl_6GeO_4$, and $Cs_{10}Tl_6SnO_3$ - First Oxotetrelate Thallides, Double Salts Containing "Hypoelectronic" Tl_6^{6-} Clusters. *Z. Anorg. Allg. Chem.* **2011**, *637* (9), 1163-1168. DOI: 10.1002/zaac.201000358.
(32) Kaskel, S.; Corbett, J. D. Synthesis and structure of $K_{10}Tl_7$: The first binary trielide containing naked pentagonal bipyramidal Tl_7 clusters. *Inorg. Chem.* **2000**, *39* (4), 778-782. DOI: 10.1021/ic991168s.
(33) Li, B.; Corbett, J. D. $Na_9K_{16}Tl_{\sim 25}$: A new phase containing naked icosahedral cluster fragments Tl_9^{9-}. *J. Clust. Sci.* **2008**, *19* (2), 331-340. DOI: 10.1007/s10876-007-0156-3.
(34) Huang, D. P.; Dong, Z. C.; Corbett, J. D. $Na_{12}K_{38}Tl_{48}Au_2$: A metallic zintl phase with naked icosahedral fragments Tl_7^{7-} and Tl_9^{9-} plus Au^-. *Inorg. Chem.* **1998**, *37* (22), 5881-5886. DOI: 10.1021/ic9808293.
(35) Vegard, L. Die Konstitution der Mischkristalle und die Raumfüllung der Atome. *Z. Phys.* **1921**, *5*, 17-26. DOI: 10.1007/BF01349680.
